# Exploitation of host U2AF1 and U2AF2 splicing factors facilitates mosquito-borne orthoflavivirus infection across species

**DOI:** 10.1093/nar/gkag713

**Published:** 2026-07-17

**Authors:** Changbai Huang, Yiquan Cai, Cancan Chen, Xuanfeng Zhu, Yingan Liang, Yingrui Luo, Qinyu Peng, Yi Wang, Chao Yang, Ming Li, Chao Liu, Ping Zhang

**Affiliations:** Key Laboratory of Tropical Diseases Control (Sun Yat-sen University), Ministry of Education, Guangzhou 510080, China; Department of Immunology and Microbiology, Zhongshan School of Medicine, Sun Yat-sen University, Guangzhou 510080, China; Key Laboratory of Tropical Diseases Control (Sun Yat-sen University), Ministry of Education, Guangzhou 510080, China; Department of Parasitology, Zhongshan School of Medicine, Sun Yat-sen University, Guangzhou 510080, China; Department of Pathology, First Affiliated Hospital of Sun Yat-sen University, Guangzhou 510080, China; Key Laboratory of Tropical Diseases Control (Sun Yat-sen University), Ministry of Education, Guangzhou 510080, China; Department of Immunology and Microbiology, Zhongshan School of Medicine, Sun Yat-sen University, Guangzhou 510080, China; Key Laboratory of Tropical Diseases Control (Sun Yat-sen University), Ministry of Education, Guangzhou 510080, China; Department of Immunology and Microbiology, Zhongshan School of Medicine, Sun Yat-sen University, Guangzhou 510080, China; Key Laboratory of Tropical Diseases Control (Sun Yat-sen University), Ministry of Education, Guangzhou 510080, China; Department of Immunology and Microbiology, Zhongshan School of Medicine, Sun Yat-sen University, Guangzhou 510080, China; Key Laboratory of Tropical Diseases Control (Sun Yat-sen University), Ministry of Education, Guangzhou 510080, China; Department of Immunology and Microbiology, Zhongshan School of Medicine, Sun Yat-sen University, Guangzhou 510080, China; Clinical Research Laboratory, Affiliated Hangzhou Xixi Hospital, Zhejiang Chinese Medical University, Hangzhou 310023, China; Department of Neurosurgery, First Affiliated Hospital of Sun Yat-sen University, Guangzhou 510080, China; Key Laboratory of Tropical Diseases Control (Sun Yat-sen University), Ministry of Education, Guangzhou 510080, China; Department of Parasitology, Zhongshan School of Medicine, Sun Yat-sen University, Guangzhou 510080, China; Key Laboratory of Tropical Diseases Control (Sun Yat-sen University), Ministry of Education, Guangzhou 510080, China; Department of Immunology and Microbiology, Zhongshan School of Medicine, Sun Yat-sen University, Guangzhou 510080, China; Key Laboratory of Tropical Diseases Control (Sun Yat-sen University), Ministry of Education, Guangzhou 510080, China; Department of Immunology and Microbiology, Zhongshan School of Medicine, Sun Yat-sen University, Guangzhou 510080, China

## Abstract

To identify novel host factors essential for orthoflavivirus replication, we performed proteomic profiling of endoplasmic reticulum fractions isolated from cells infected with Dengue virus or Zika virus (ZIKV). Among the enriched proteins, the splicing factor U2AF2 and its heterodimeric partner U2AF1 were found to be critical for efficient viral infection, functioning at the viral protein synthesis stage. The arginine/serine-rich (RS) domain and the first zinc knuckle (Zn1) of U2AF1, as well as nearly all domains except the RS domain of U2AF2, were essential for their proviral function. Disruption of the U2AF1–U2AF2 interaction potently suppressed ZIKV infection. U2AF1 and U2AF2 partially localize to the cytoplasm, and notably, the cytoplasmic U2AF2 was predominantly a truncated form that was sufficient to support viral replication. Both U2AF1 and U2AF2 bind to viral RNA, with U2AF1 binding being dependent on U2AF2. Interestingly, *trans*-complementation of the *Aedes aegypti* homolog *u2af38* into U2AF1-knockout cells restored ZIKV replication, whereas expressing *u2af50* in U2AF2-knockdown cells did not. However, knockdown of either *u2af38* or *u2af50* significantly inhibited the flavivirus replication in mosquitoes. Together, these findings reveal that flaviviruses co-opt host splicing factors in both human cells and mosquitoes, underscoring a conserved cross-species mechanism of viral exploitation.

## Graphical Abstract

**Graphical Abstract:**
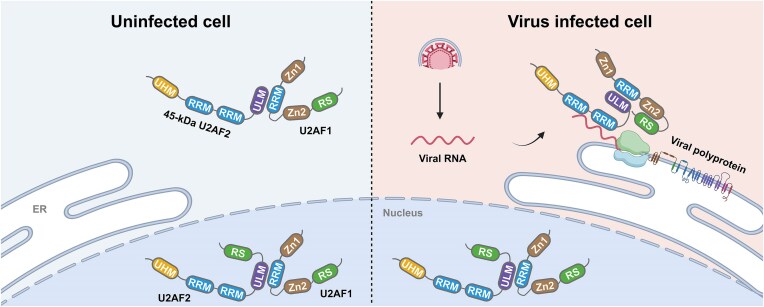

## Introduction

Mosquito-borne orthoflaviviruses, including Zika virus (ZIKV) and Dengue virus (DENV), present a significant and ongoing threat to global public health [1, 2]. Infection caused by these viruses can lead to a wide spectrum of clinical manifestations, ranging from hemorrhagic fever, arthritis, and neurological complications (encephalitis and meningitis) to severe, life-threatening outcomes. In recent decades, the incidence caused by orthoflaviviruses has grown dramatically and cumulatively infect over 390 million people annually, leading to 96 million symptomatic cases and 10 000 deaths worldwide [3]. Nonetheless, there are still no antiviral therapies specific against diseases caused by orthoflavivirus infection [1, 4].

The genome of mosquito-borne orthoflaviviruses is a single-stranded, positive-sense RNA featuring a 5′ cap and a single open reading frame (ORF) flanked by highly structured 5′- and 3′-untranslated regions (UTRs) [5]. The ORF encodes three structural and seven non-structural proteins, which collectively support the viral life cycle and host modulation, whereas the UTRs coordinate viral translation, replication, and regulation of the host innate immune response [3]. The life cycle of these viruses begins with the recognition and binding of cellular receptors, triggering internalization and membrane fusion [6]. Subsequently, the viral genomic RNA is released into the cytoplasm, where it serves as a template for cap-dependent or IRES-like translation [7], producing a polyprotein. This polyprotein is then cleaved by host and viral proteases into individual proteins, followed by viral RNA replication. Newly synthesized viral RNA and structural proteins assemble to form progeny virions. Finally, the progeny virions undergo maturation in the Golgi apparatus before being secreted from the cell [8].

As obligate intracellular pathogens, orthoflaviviruses rely entirely on host cell organelles for replication [9]. Particularly, the endoplasmic reticulum (ER) serves as a central hub in the orthoflavivirus infection, supporting viral protein synthesis, polyprotein processing, RNA replication, and virion assembly [10]. Notably, orthoflaviviruses extensively remodel ER membranes to create specialized organelle-like structures known as replication organelles (ROs) [11–13]. ROs serve as dedicated platforms that concentrate both viral components and hijacked host factors to facilitate efficient viral RNA replication [11, 14, 15]. Current research has identified various ER-resident host factors that support different stages of the flavivirus replication cycle. For example, adenosylhomocysteinase enhances ZIKV immune evasion by reducing S-adenosylhomocysteine level and thereby promoting m6A methylation of viral RNA [16]; inositol-requiring enzyme 1α (IRE1α) stimulates lipid droplet formation and viral replication via XBP1/stearoyl-CoA desaturase 1 [17]; Aldo-keto reductase AKR1C3 modulates ZIKV capsid protein stability, improving virion assembly efficiency [16]; and thioredoxin reductase 1 facilitates proper folding of viral proteins and regulates mature virion secretion [16].

In this study, we sought to find new host factors that are recruited to the ER by orthoflaviviruses and functionally regulate viral replication by ER proteomic profiling of virus-infected cells. Among the infection-induced ER-enriched proteins, we identified U2 snRNP (small nuclear RNP) auxiliary factor 65 kDa subunit (U2AF65, also known as U2AF2) as a critical host factor. We extensively explored the underlying mechanism by which U2AF2 and its binding partner the 35 kDa subunit (U2AF35, also known as U2AF1) promote orthoflavivirus infection. U2AF2 and U2AF1 are primarily localized in the nucleus, where they play essential roles in pre-mRNA splicing [18, 19]; they can also traffic through the cytosol [20]. Structurally, U2AF2 consists of an N-terminal arginine/serine-rich (RS) domain, a UHM (U2AF homology motif) ligand motif (ULM) that mediates protein interactions, two RNA recognition motifs (RRM1 and RRM2), and a C-terminal UHM [21, 22]. The U2AF1 protein contains two zinc knuckle motifs (Zn1 and Zn2) flanking an RRM domain (which also functions as a UHM domain), followed by a C-terminal RS domain [23]. U2AF2 and U2AF1 forms a heterodimer involved in regulating the splicing of pre-mRNA [24]. Given their central role in RNA processing, it is not surprising that U2AF1 and U2AF2 are co-opted by various viruses, such as human immunodeficiency virus type 1 (HIV-1) [25], human papillomavirus (HPV) [26], influenza A virus (IAV) [27], adeno-associated viruses [28], and avian reovirus [29]. To be noted, during our investigation, Song *et al*. reported U2AF2 as a regulatory factor in Japanese encephalitis virus (JEV) replication [30]. However, key mechanistic questions remain unresolved: (i) At which specific stage of the viral life cycle does U2AF2 regulate? (ii) Which specific domain(s) of U2AF2 mediate its cytoplasmic localization and biological activity? (iii) Is U2AF2 binding partner U2AF1 recruited to support viral replication? (iv) Do U2AF2 and U2AF1 similarly promote *in vivo* viral replication in mosquito vectors?

To address these questions, we applied loss-of-function approaches in *in vitro* (cell culture) and *in vivo* (mosquito) models. We demonstrated that U2AF1 and U2AF2 function at the protein synthesis step of ZIKV, and mapped the essential domains of U2AF1 and U2AF2 mediating their proviral function. Our data revealed that pharmacological inhibition of UHM-mediated protein interactions using 7,8-dihydroxyperphenazine or introduction of a point mutation (W92A) in U2AF2 significantly suppresses viral infection. Furthermore, we analyzed the subcellular localizations of U2AF1 and U2AF2, and discovered that in the cytoplasm U2AF2 is mainly present as a truncated form (45 kDa), which is sufficient to support flaviviral replication. In addition, we comprehensively determined the contributions of U2AF1 and U2AF2 to their association with viral RNA, and revealed that the U2AF1-RNA binding is dependent on U2AF2. Finally, our study explored role of u2af38 and u2af50, the *Aedes aegypti* homologs of U2AF1 and U2AF2, in the ZIKV and DENV2 infection in both human cells and mosquitoes. This work establishes that flaviviruses exploit two splicing factors in both host and mosquito vector, revealing a conserved mechanism of viral exploitation across species.

## Materials and methods

### Cells and mosquitoes

Human hepatoma cells (Huh7.5) cells were provided by Prof. Yiping Li (Sun Yat-sen University). Huh7.5 cells, human lung carcinoma epithelial cells [A549, American Type Culture Collection (ATCC), CCL-185], human glioblastoma cells (SNB19, ATCC CRL-2219), and human embryonic kidney cells (293 T, ATCC, CRL-3216) were maintained in Dulbecco’s modified Eagle medium (DMEM, Gibco) supplemented with 10% fetal bovine serum (FBS, Gibco) at 37°C in an incubator with 5% CO_2_. African green monkey kidney cells (Vero, ATCC, CCL-81) and baby hamster kidney cells (BHK-21, ATCC, CCL-10) were maintained in DMEM supplemented with 5% FBS at 37°C in an incubator with 5% CO_2_. The media were added with 100 units/ml of streptomycin and penicillin (Invitrogen).

*Aedes aegypti* mosquitoes (the Rockefeller strain) were reared in a sugar solution at 28°C and 80% humidity following published standard rearing procedures. Female mosquitoes were selected to further investigation.

### Viruses, virus infection, and virus titration

ZIKV strain H/PF/2013 (GenBank: KJ776791), DENV2 (DENV2 16681), and JEV (14-14-2 vaccine strain) were obtained from Guangzhou Centers for Disease Control and propagated in Vero cells or C6/36 cells. SARS-CoV-2 ΔN trVLP were kindly provided by Prof. Rong Zhang (Fudan University). EMCV and SFV were kindly provided by Prof. Rongbin Zhou (University of Science and Technology of China) and Prof. Xi Zhou (Wuhan Institute of Virology), and propagated in HeLa and C6/36 cells, respectively. HSV-1 was propagated in Vero cells. All viral stocks were clarified by centrifugation, aliquoted, and stored at −80°C until use.

Cells were infected with viruses at a multiplicity of infection (MOI) of 3 to ensure that most cells were infected unless specifically indicated. For ZIKV, DENV2, JEV, and SFV, supernatants were harvested depending on the experiment endpoints, and cell debris was removed by centrifugation at 185 × *g* for 5 min and filtration. For EMCV and HSV-1, both supernatants and cells were collected, followed by three cycles of freezing-and-thawing and centrifugation at 185 × *g* for 5 min.

Virus titers were determined by standard plaque assay. Serial 10-fold dilutions of each sample were prepared, and 100 μl/well of the diluted virus were applied. The cells were cultured at 37°C for 1 h and intermittently rocked every 15 min to allow the adsorption of virions. The incubation medium was removed and cultured in the mixture of 2× MEM (Invitrogen) and isopycnic 2% low melting point agarose (Sangon Biotech; 1:1) or 2% methylcellulose (1:1) (Sigma–Aldrich). Visible plaques were counted at 2–3 days (SFV, HSV-1, EMCV), 4–5 days (ZIKV, JEV), or 5–7 days (DENV2). Cells were fixed with 10% formaldehyde and plaques were visualized by staining with 1% crystal violet.

### ER isolation and mass spectrum

Huh7.5 cells were infected with ZIKV or DENV2 (strain 16681) at an MOI of 1. At 24 h post infection (p.i.), the ER fraction was isolated using an ER Enrichment Kit (ER-036, Invent) according to the manufacturer’s instructions. Uninfected Huh7.5 cells served as a negative control. The samples were then subjected to sodium dodecyl sulfate–polyacrylamide gel electrophoresis (SDS–PAGE), and each entire gel lane was excised and analyzed by liquid chromatography-mass spectrometry.

Raw mass-spectrometry data were processed with MaxQuant software versions 1.5.6.262 using the built-in Andromeda search engine to search against the human proteome containing forward and reverse sequences. Search results were filtered with a false discovery rate of 0.01 for peptide and protein identifications.

### Plasmid construction

Oligonucleotide sequences of single guide RNAs targeting individual genes were listed in Supplementary Table S1. Oligonucleotides were annealed and inserted into plasmid vector lentiCRISPR v2 (Addgene, #52961). The pLKO.1-TRC plasmid (Addgene, #10878) was utilized to construct U2AF2-shRNA, or control-shRNA (shCtrl, scramble sequence) to generate U2AF2-knockdown (KD) cells as previously described [31].

Fragments of full-length (FL) of U2AF1 rescue mutant or truncates with deleted zinc finger domain (ΔZn1 and ΔZn2), RNA recognize motif (ΔRRM), or serine-arginine rich domain (ΔRS) were amplified by polymerase chain reaction (PCR) using complementary DNA (cDNA) template from A549 cells. Fragments of FL of U2AF2 rescue mutant or truncates with deleted serine-arginine rich domain (ΔRS), UHM ligand motif (ΔULM), RNA recognize motif (ΔRRM), or U2AF homology motif (ΔUHM) were amplified by PCR using cDNA template from A549 cells. Primer sequences were listed in Supplementary Table S2. PCR fragments were purified and cloned into lentiviral vector pLV-EF1α-IRES-blasticidin (Addgene, #85133). Positive clones were verified by DNA sequencing.

The 5′ and 3′ UTRs of ZIKV were PCR-amplified from a ZIKV replicon plasmid pFK-SGR (a gift from Prof. Gang Long, Fudan University) [32] using the primers listed in Supplementary Table S2. The PCR fragments were digested with EcoRI and then self-ligated to generate pFK-5′UTR and pFK-3′UTR, respectively. Positive clones were verified by DNA sequencing.

### Generation of U2AF1 knockout and U2AF2 knockdown cells

U2AF1 knockout (KO) cell clones were generated using Clustered Regularly Interspaced Short Palindromic Repeats (CRISPR)/CRISPR-associated protein 9(Cas9). Single-guide RNAs (sgRNAs) targeting U2AF1 were cloned into lentiCRISPR v2. Sequences of sgRNAs are listed in Supplementary Table S1. Short hairpin RNAs (shRNAs) targeting U2AF2 were cloned into pLKO.1-TRC. Sequences of shRNAs are listed in Supplementary Table S1. Lentiviruses were packaged in 293T cells. LentiCRISPR v2 containing single sgRNA or pLKO.1-TRC containing single shRNA, along with pSPAX2 (Addgene, 12260) and pVSVG (Addgene, 12259), were introduced into 293T cells using FuGENE HD Reagent (Promega). After 2 days, culture supernatants were passed through a 0.45-μm filter and used for gene transduction. A549 cells were transduced with lentiviruses and selected by puromycin (Invivogen, 1 μg/ml). Genomic DNA of U2AF1^KO^ cell was extracted using a genomic DNA extraction kit (Bioteke) and then the PCR products were subjected to Nanopore sequencing.

### Generation of full length, truncated, or point mutant form U2AF1/2 expressing cells

Lentiviruses carrying gene expressing full length or truncated form of U2AF1 or U2AF2 were packaged in 293T cells. 293T cells were transfected with plasmids (pLV-res-sgU2AF1#1, pLV-U2AF1-ΔZn1, pLV-U2AF1-ΔRRM, pLV-U2AF1-ΔZn2, pLV-U2AF1-ΔRS, pLV-u2af38, pLV-res-shU2AF2#1, pLV-U2AF2-ΔRS, pLV- U2AF2-ΔULM, pLV-U2AF2-ΔRRM, pLV-U2AF2-ΔUHM, pLV-U2AF2-D128A, pLV-U2AF2-Δ64–84, pLV-U2AF2-Δ141–148, pLV-U2AF2-Δ84, pLV-U2AF2-Δ148, pLV-U2AF2-W92A, or pLV-u2af50) and two packaging plasmids pSPAX2 and pMD2.G using FuGENE HD transfection reagent (Promega). Supernatants were collected at 2 days post-transfection (p.t.) and passed through a 0.45-μm filter. Subsequently, U2AF1^KO^ cells were transduced with lentiviruses carrying full length of U2AF1, ΔZn1, ΔRRM, ΔZn2, or ΔRS. U2AF2^KD^ cells were transduced with lentiviruses carrying gene expressing full length of U2AF2, ΔRS, ΔULM, ΔRRM, or ΔUHM. Stable cells were generated by selection and expansion in the presence of 15 μg/ml blasticidin (Invivogen) for 7–10 days.

### Double-stranded RNA synthesis

The genes of interest were PCR-amplified using gene-specific primers designed to amplify fragments of ~500 bp from the 3′ region. Each primer pair included a T7 promoter sequence incorporated into both the forward and reverse primers (Supplementary Table S4). PCR amplification was performed using a 2× Phanta Max Master Mix (Vazyme, P515-01). The PCR fragments were then purified and used for subsequent double-stranded RNA (dsRNA) synthesis. dsRNA molecules were generated using the MEGAscript™ T7 Transcription Kit (Thermo Fisher Scientific, AM1334). Following the manufacturer’s instructions, a 20 μl *in vitro* transcription reaction containing RNA Polymerase Enzyme Mix, NTPs, template DNA, and 10× Reaction Buffer was incubated at 37°C for 2–4 h. Next, 1 μl TURBO DNase was added, and the mixture was incubated for an additional 15 min at 37°C to degrade residual template DNA. RNA was subsequently purified by LiCl precipitation. Prior to transcription, Sanger sequencing was performed to verify the sequence fidelity of the template DNA. The formation and integrity of annealed dsRNA were assessed using agarose gel electrophoresis and spectrophotometry.

### Gene silencing and viral infection in mosquitoes

Female mosquitoes were first anesthetized on a cold tray, and 1 μg/300 nl of dsRNA was microinjected into their thoraxes. A total of 10 M.I.D50/300 nl virus was intrathoracically microinjected into per mosquito 3 days later. The gene silencing efficiency was assessed by reverse transcription quantitative polymerase chain reaction (RT-qPCR). The primers used for dsRNA synthesis and gene detection are shown in Supplementary Tables S3 and S4.

### CCK8 assay

Cell Counting Kit-8 (CCK8) was purchased from MCE. Cells were seeded in 12-well plates, and 24 h later, 80 μl of CCK-8 reagent was added to each well, followed by incubation for 1 h. Absorbance was measured at 450 nm using a BioTek microplate reader.

### Antibodies

Primary antibodies used in western blot and immunofluorescence were listed in Supplementary Table S5. Secondary antibodies include IRDye 800 CW-conjugated anti-rabbit IgG, IRDye 680 CW-conjugated anti-mouse IgG (LI-COR), and horseradish peroxidase-conjugated anti-mouse IgG (CST). Secondary antibodies used in immunofluorescence assay included goat anti-rabbit IgG secondary antibody (Alexa Fluor 488) and goat anti-mouse IgG secondary antibody (Alexa Fluor 647) from Invitrogen.

### Western blot

Cells were lysed in RIPA lysis buffer [pH 7.4; 50 mM Tris–HCl, 0.5% (v/v) NP-40, 1% Triton X-100, 150 mM NaCl, 1 mM ethylenediaminetetraacetic acid (EDTA), 1 mM phenylmethylsulfonyl fluoride (PMSF), 1% protease inhibitor cocktails (Sigma–Aldrich), 1 mM Na_3_VO_4_, and 1 mM NaF]. Proteins were separated on SDS–PAGE and transferred onto nitrocellulose membranes. The membranes were blocked in phosphate buffered saline (PBS) plus Tween 20 with 5% bovine serum albumin (BSA; New England Biolabs) and incubated with the indicated primary antibodies at 4°C overnight. IRDye 800 CW-conjugated anti-rabbit IgG (LI-COR), IRDye 680 CW-conjugated anti-mouse IgG (LI-COR), or horseradish peroxidase-conjugated secondary antibodies (Bio-Rad) served as secondary antibodies. Detection was performed according to the manufacturer’s protocols.

### RNA interference

The sequences of the siRNAs targeting human *U2AF1* and *U2AF2* were 5′-GAAAGTGTTGTAGTTGATTGA-3′ and 5′- ACCCAACTACCTGAACGATGA-3′, respectively. A control siRNA with scrambled sequence was used as a negative control (siNC). Transfection was carried out with 16 nmol of siRNAs by using Lipofectamine 2000 Reagent (Invitrogen) according to the manufacturer’s instruction. At 48 h p.t., cells were harvested for further analysis.

### RT-qPCR

Total RNAs were reverse transcribed using HI Script Q RT SuperMix (Vazyme). The cDNA was used as the template for RT-qPCR. The RT-qPCR was performed using SYBR Select Master Mix for CFX (Applied Biosystems) and a Bio-Rad CFX96 machine. The RT-qPCR data were analyzed using SDS software (Applied Biosystems). The mRNA level of *β-actin* was measured as an internal control. The primers used for RT-qPCR are listed in Supplementary Table S3.

### Virus entry assay

For the virus binding assay, cells were pre-chilled on ice for 10 min prior to ZIKV infection. After two washes with ice-cold PBS, cells were incubated with ZIKV at an MOI of 3 in ice-cold DMEM supplemented with 2% FBS, followed by a 45-min incubation on ice to allow virion binding. Subsequently, the cells were washed five times with PBS and lysed in TRIzol reagent (Thermo Fisher Scientific, Cat. #15596018) for total RNA extraction. For the virus internalization assay, cells were inoculated with ZIKV at an MOI of 3 at 37°C for 45 min. After the culture medium was removed and the cells were washed three times with PBS, 400 μg/ml proteinase K was added, and the mixture was incubated on ice for 45 min to eliminate uninternalized surface viruses. Following three additional PBS washes, cells were lysed with TRIzol reagent for RNA extraction. RT-qPCR was then performed to quantify viral RNA levels, with β-actin serving as the internal control.

### Replicon assay

The ZIKV replicon plasmids pFK-SGR and pFK-SGR-GDD were kindly provided by Prof. Gang Long at Fudan University [32]. Briefly, the pFK-SGR, pFK-SGR-GDD, pFK-5′UTR, and pFK-3′UTR were linearized with MluI (New England Biolabs), and extracted by phenol–chloroform and precipitated with sodium acetate. The linearized DNA template was transcribed using mMESSAGEmMACHINE® T7 kit (Thermo Fisher Scientific, AM1334) according to the manufacturer’s protocol. The product of transcription was purified by lithium chloride (LiCl) precipitation. Replicon RNAs (0.2 μg/well) were transfected into cells using Lipofectamine® 2000 reagent (Invitrogen). Cells were washed with PBS and lysed using passive lysis buffer (Promega) at indicated time points. Luciferase activities were measured using a GLOMAXTM 96 microplate luminometer (Promega).

### Polysome fractionation assay

Control, U2AF1^KO^, and U2AF2^KD^ cells were treated with 100 mg/ml cycloheximide at 37°C for 5 min. Whole-cell extracts were prepared, layered onto a 10%–50% continuous sucrose gradient, and centrifuged at 39 000 rpm in a Beckman SW-41Ti rotor for 3 h at 4°C. Twelve fractions were collected sequentially from the top of the gradient. RNA was then extracted from each fraction and analyzed by RT-qPCR.

### Subcellular fractionation assay

Cells were washed twice with PBS and lysed in cytoplasmic extract buffer [10 mm HEPES, 60 mm KCl, 1 mm EDTA, 0.075% (v/v) Nonidet *P*-40, 1 mm DTT, and 1 mm PMSF]. The mixture was spun at 14 000 rpm for 5 min to separate the cytoplasmic extract (supernatant). The precipitate was washed with cytoplasmic extract buffer and then lysed in nuclear extract buffer [20 mm HEPES, 420 mm NaCl, 10 mm KCl, 1 mm EDTA, 1 mm PMSF, and 20% (v/v) glycerol]. The mixture was spun at 14 000 rpm for 5 min to separate the nuclear extract (supernatant).

### Immunofluorescence microscopy

Cells were fixed with 4% PFA after three washes with PBS and permeabilized with 0.02% Triton-X 100 for 15 min. Then, cells were blocked with 5% BSA for 30 min, followed by incubation with primary antibody at 4°C overnight. After washes, cells were incubated with Alexa Fluor 647-conjugated goat anti-mouse-IgG (Invitrogen) or Alexa Fluor 488-conjugated anti-rabbit-IgG (Invitrogen) in PBS at room temperature for 1 h. Cells were then stained with TRITC Phalloidin (Yeasen) diluted in PBS for 45 min, followed by incubation with DAPI (Invitrogen) diluted in PBS for 20 min. Fluorescence images were acquired with a Nikon C2 microscope and analyzed with the NIS Elements software.

### RNA immunoprecipitation assay

The ZIKV- infected or GDD replicon RNA-transfected cells were lysed in RNA immunoprecipitation (RIP) lysis buffer [100 mM KCl, 5 mM MgCl_2_, 0.5% (v/v) NP-40, 10 mM HEPES, 1 mM PMSF, 1% protease inhibitor cocktails, and RNase inhibitor), and the lysates were pretreated with protein A/G agarose (Calbiochem). Immunoprecipitation was carried out using anti-U2AF2, anti-FLAG, or control mouse IgG. Immune complexes were precipitated with protein A/G agarose. Viral RIP was performed using TRIzol reagent (Invitrogen) according to the manufacturer’s protocol. Viral RNA levels were analyzed by RT-qPCR (primer sequences are listed in Supplementary Table S3).

### Statistical analysis

All experiments were independently repeated at least three times. Comparisons between two groups were performed using ANOVA, ANOVA with Dunnett’s multiple comparison test, or an unpaired, two-tailed Student’s *t*-tests. Graphs were generated using Graph Pad Prism 8.0 software. *P*-values of .05 or lower were considered to be statistically significant.

## Results

### Identification of endoplasmic reticulum-enriched host factors regulating flavivirus replication

**Figure 1.**
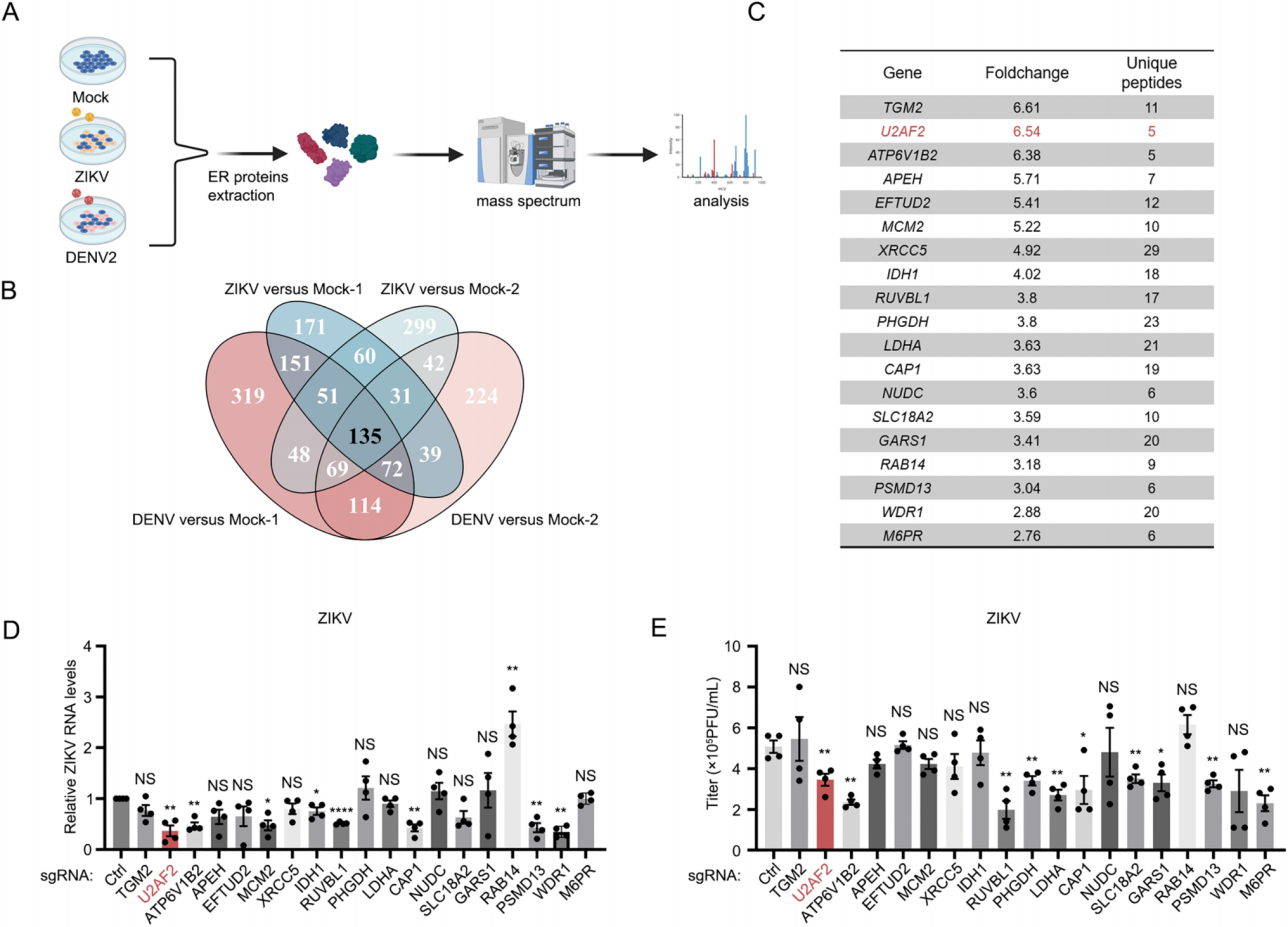
A mass spectrometry screen identifies proteins enriched in ER during flavivirus infection. (**A**) Diagram summarizing the screening strategy. Created in BioRender (Huang C., 2026, https://BioRender.com/ytsah68). (**B**) Venn diagram showing shared peptides between the ZIKV- and DENV2-infected Huh7.5 cells. (**C**) Top-ranking genes showing the gene name, fold change, and number of enriched unique peptides. (**D, E**) Gene-edited Huh7.5 bulk cells were generated by lentivirus-mediated transduction and drug selection. Cells were infected with ZIKV for 24 h, and the levels of viral RNA and titers were determined by RT-qPCR and plaque assay, respectively. Data are presented as mean ± standard error of mean (SEM) from three biologically independent experiments. Statistical significance was determined using one-way ANOVA with Dunnett’s multiple comparison test (*P < .05, **P < .01, ****P < .0001, and NS, not significant).

As flavivirus biosynthesis primarily occurs in the ER, we hypothesized that numerous host proteins are recruited and concentrated in the ER to facilitate viral infection. To test this, we isolated the ER fractions of mock- and virus-infected cells and identified potential enriched proteins by MS (mass spectrometry)-based proteomic analysis. Human hepatoma Huh7.5 cells, which are highly permissive to flaviviral replication [33], were infected with mock or flaviviruses (ZIKV or DENV2) at an MOI of 1. At 48 h p.i., the ER fractions were extracted using an ER isolation kit and submitted for MS analysis (Fig. 1A). Effective enrichment of the ER was validated by western blot, showing high levels of the ER marker calnexin and minimal contamination from nuclear and endosomal protein markers (Supplementary Fig. S1A). Proteomic analysis revealed 277 proteins enriched in ZIKV-infected cells, 390 in DENV2-infected cells, and 135 shared between both viral infections (Fig. 1B and Supplementary Table S6). Importantly, the identified proteins included 25 previously characterized flavivirus host factors (such as AP1G1 [34, 35], IPO7 [36], and MOV10 [37]), demonstrating the effectiveness of our screening methodology.

To assess role of these ER-enriched proteins in flaviviral infection, we employed a loss-of-function screen utilizing CRISPR/Cas9 gene editing technique (Fig. 1C). The screening panel comprised 19 top-ranking genes that were not previously associated with flaviviruses. Bulk Huh7.5 cells carrying sgRNA targeting each gene or control sgRNA were generated by lentivirus-mediated transduction. The efficiencies of sgRNA-mediated gene editing were confirmed by real-time PCR (Supplementary Fig. S1B). Then, Huh7.5 cells were infected with ZIKV, and harvested at 24 h p.i. for measurement of viral RNA levels and titers. The RT-qPCR and plaque assay data showed that knockout of *U2AF2, ATP6V1B2, RUVBL1, CAP1*, or *PSMD13* resulted in significant reductions of ZIKV RNA levels and titers (Fig. 1D and E). Given that U2AF2 was among the most highly enriched proteins in the virus-infected ER and exhibited significant proviral activity, we focused our investigations on its role in the flavivirus infection.

### U2AF2 and U2AF1 enhance viral infection in a virus-specific manner

**Figure 2.**
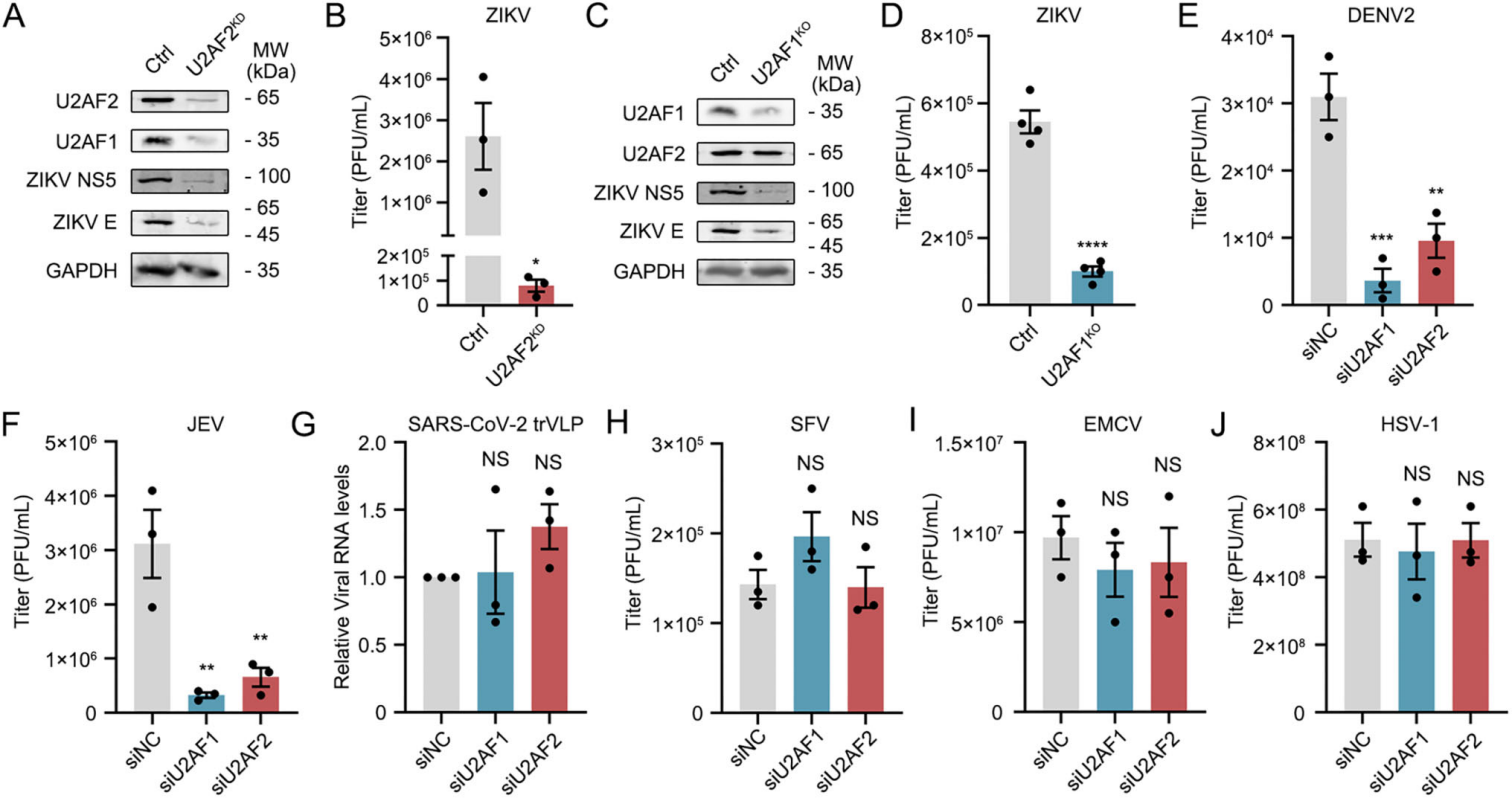
U2AFs specifically facilitate flavivirus infection. (**A**–**D**) Control, U2AF2^KD^, and U2AF1^KO^ A549 cells were infected with ZIKV at an MOI of 3. At 24 h p.i., the cells and supernatants were collected for western blot using antibodies against U2AF2, U2AF1, ZIKV E, ZIKV NS5, or GAPDH and plaque assay. Representative images of three independent experiments are shown. The data are shown as mean ± SEM from three biologically independent experiments. Statistical significance was determined using two-tailed unpaired *t*-test (*P < .05, and ****P < .0001). (E–J) Viral replication level. A549 cells were transfected with siRNA against control (siNC), U2AF1 (siU2AF1), or U2AF2 (siU2AF2). At 48 h post transfection, cells were infected with DENV2 (**E**), JEV (**F**), SARS-CoV-2 trVLP (**G**), SFV (**H**), EMCV (**I**), or HSV-1 (**J**) at an MOI of 3. At 24 h p.i., cells and supernatants were collected for plaque assay. SARS-CoV-2 trVLP-infected cells were collected for RT-qPCR. Data are presented as mean ± SEM from three biologically independent experiments. Statistical significance was determined using one-way ANOVA with Dunnett’s multiple comparison test (**P < .01, ***P < .001, and NS, not significant).

To assess the role of U2AF2 in flavivirus infection, we established stable knockdown cell pools using RNA interference (RNAi) strategy as knockout of U2AF2 is lethal. A549 cells were infected with lentivirus carrying shRNA targeting U2AF2, and selected by puromycin for a week, followed by virus infection. The U2AF2 level was successfully downregulated in the U2AF2 knockdown cells (U2AF2^KD^). Of note, the U2AF1 level in the U2AF2^KD^ cells was simultaneously downregulated (Fig. 2A). As expected, the depletion of U2AF2 led to significant reduction of ZIKV non-structural protein NS5 and envelope (E) levels, as well as viral titers (Fig. 2A and B).

Considering both U2AF2 and U2AF1 levels were reduced by U2AF2 silencing, we then determined role of U2AF1 in the viral replication by generating U2AF1 knockout (U2AF1^KO^) cells using CRISPR/Cas9 editing technique. DNA sequencing data showed that the flanking sequences around the sgRNA-targeted site of *U2AF1* were either partially deleted (22 bp and 25 bp deletions) or remained intact, indicating that the U2AF1^KO^ cells are not complete knockouts (Supplementary Fig. S2A), therefore the growth rates of U2AF1^KO^ cells were comparable to control cells (Supplementary Fig. S2B). The U2AF1 deficiency impaired the viral protein levels (NS5 and E) and titer without altering U2AF2 levels (Fig. 2C and D), demonstrating a role of U2AF1 in ZIKV replication. Furthermore, we examined role of U2AF1/2 in another two permissive cell lines (Huh7.5 and human glioblastoma SNB19 cells) using RNAi technique. The knockdown efficacies of siRNAs targeting U2AF1 or U2AF2 were validated by RT-qPCR and western blot (Supplementary Fig. S3A and B). In all tested cells, knockdown of U2AF1 or U2AF2 alone led to a significant reduction of ZIKV RNA levels and titers (Supplementary Fig. S3B and C), suggesting that the proviral effects of U2AF1 and U2AF2 are not cell specific.

Next, we investigated the impact of U2AF1 and U2AF2 depletion on the infection of other viruses, including five positive-sense RNA viruses [DENV2, JEV, SARS-CoV-2, Semliki Forest virus (SFV), and encephalomyocarditis virus (EMCV)] and one DNA virus [herpes simplex virus 1 (HSV-1)]. Using siRNA-mediated knockdown in A549 cells, U2AF1 or U2AF2 were successfully depleted, and their loss significantly reduced viral titers of DENV2 and JEV but had no effect on SARS-CoV-2, SFV, EMCV, or HSV-1 (Fig. 2E–J). These results suggest that U2AF proteins specifically promote the replication of flaviviruses.

### Identification of U2AF1 and U2AF2 domains mediating their proviral effect

**Figure 3.**
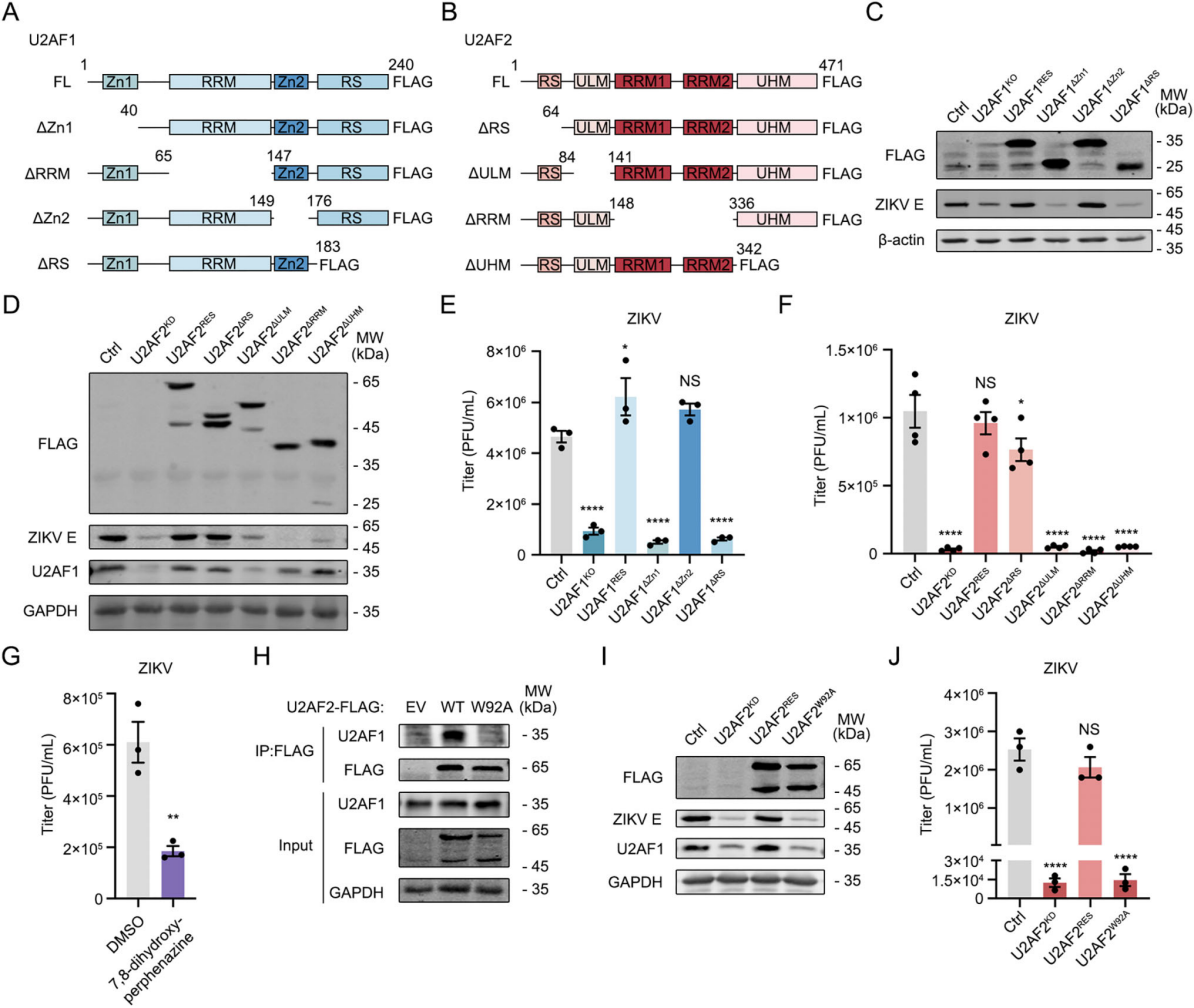
Mapping of U2AF1 and U2AF2 domains mediating their proviral effect. (**A, B**) Schematic representation of FL and truncated U2AFs. (**C–F**) ZIKV replication levels. Control (transduced with backbone plasmids pLKO.1-SCR and pLVEF1a-IRES-Blast-85133), U2AF1^KO^, U2AF1^RES^, U2AF1^ΔZn1^, U2AF1^ΔZn2^, and U2AF1^ΔRS^ cells were infected with ZIKV at an MOI of 3. At 24 h p.i., cells and supernatants were harvested for western blot (**C**) and plaque assay (**E**). Control (transduced with backbone plasmids lentiCRISPR v2 and pLVEF1a-IRES-Blast-85133), U2AF2^KD^, U2AF2^RES^, U2AF2^ΔRS^, U2AF2^ΔULM^, U2AF2^ΔRRM^, and U2AF2^ΔUHM^ cells were infected with ZIKV at an MOI of 3. At 24 h p.i., cells and supernatants were harvested for western blot (**D**) and plaque assay (**F**). Data are shown as mean ± SEM from three biologically independent experiments. Statistical significance was determined using one-way ANOVA with Dunnett’s multiple comparison test (*P < .05, ****P < .0001, and NS, not significant). (**G**) Impact of UHM inhibitor on ZIKV replication. Huh7.5 cells were pretreated with DMSO or 10 μM of 7,8-dihydroxyperphenazine for 1 h, followed by ZIKV infection (MOI 3). At 24 h p.i., supernatants were collected for plaque assay. (H–J) Impact of U2AF2 W92A mutation on viral replication. Co-IP assay to show the interaction between U2AF1 and WT or mutant (W92A) U2AF2 (**H**). Control, U2AF2^KO^, U2AF2^RES^, and U2AF2^W92A^ cells were infected with ZIKV at an MOI of 3. At 24 h p.i., cells and supernatants were harvested for western blot (**I**) and plaque assay (**J**). The data are shown as mean ± SEM from three biologically independent experiments. Statistical significance was determined using two-tailed unpaired *t*-test or one-way ANOVA (**P < .01, ****P < .0001, and NS, not significant).

To map the essential domains of U2AF1 and U2AF2 required for their proviral function, we constructed lentivirus-based vectors expressing FL or their truncated mutants. Truncated mutants of U2AF1 included U2AF1^ΔZn1^ lacking the N-terminal zinc finger domain (Zn) 1, U2AF1^ΔRRM^ lacking the RNA recognition motif (RRM, also acts as UHM), U2AF1^ΔZn2^ lacking the Zn2 domain, and U2AF1^ΔRS^ lacking the C-terminal serine-arginine rich (RS) domain (Fig. 3A). Truncated mutants of U2AF2 included U2AF2^ΔRS^ lacking the N-terminal RS domain, U2AF2^ΔULM^ lacking the UHM ligand motif (ULM), U2AF2^ΔRRM^ lacking both RRMs, and U2AF2^ΔUHM^ lacking the C-terminal UHM (Fig. 3B). The above vectors were utilized to establish cell lines stably expressing FL or truncated U2AF1 or U2AF2 through lentiviral transduction followed by drug selection. Then, these cells were infected with ZIKV, and at 24 h p.i., cells and supernatants were collected for measurement of viral replication levels. Western blot data confirmed that expression of FL and most truncated mutants of U2AF1 and U2AF2 were successful (Fig. 3C and D), except U2AF1^ΔRRM^ (despite different vectors were tried) (Supplementary Fig. S4A). To be noted, the observed molecular weights of U2AF1^ΔZn1^, U2AF1^ΔZn2^, and U2AF2^ΔUHM^ differed slightly from their expected values (29, 32, and 47 kDa, respectively), suggesting that these proteins might undergo post-translational modifications. *Trans*-complementation of FL U2AF1 in the U2AF1^KO^ cells or FL U2AF2 in the U2AF2^KD^ cells restored the viral E levels and titers (Fig. 3C–F). Expression of U2AF1^ΔZn2^ but not U2AF1^ΔZn1^ or U2AF1^ΔRS^ in the U2AF1^KO^ cells largely rescued the ZIKV replication levels (Fig. 3C and E), indicating that Zn1 and RS domains of U2AF1 are essential for its proviral function. Of note, the U2AF1 protein levels were largely restored in the cells expressing U2AF2^RES^, U2AF2^ΔRS^, U2AF2^ΔRRM^, and U2AF2^ΔUHM^, but not U2AF2^ΔULM^ (Fig. 3D), suggesting that ULM domain of U2AF2 is essential for stabilizing of U2AF1. In the U2AF2^KD^ cells, *trans*-complementation with all truncated forms of U2AF2 did not affect the cell viability (Supplementary Fig. S4B–D). Only expression of U2AF2^ΔRS^ restored the ZIKV replication levels, while expression of another three truncated mutants (U2AF2^ΔULM^, U2AF2^ΔRRM^, or U2AF2^ΔUHM^) showed minimal effect on viral infection (Fig. 3D and F), suggesting that the proviral effect of U2AF2 relied on nearly all functional domains except RS domain.

As ZIKV infection requires the UHM domain of U2AF2, which mediates protein–protein interaction, we hypothesized that heterodimerization of U2AF1 and U2AF2 is important for their proviral function. To test this, we utilized a pharmacological inhibitor, 7,8-dihydroxyperphenazine, that competitively interact with UHM domain [38]. Treatment of 7,8-dihydroxyperphenazine led to a significant reduction of ZIKV titers (Fig. 3G). Furthermore, we established stable cells expressing a point mutant of U2AF2 (W92A), which disrupts its interaction with U2AF1 [39]. Co-Immunoprecipitation (Co-IP) assays confirmed that the U2AF2 W92A mutation abolished its binding to U2AF1 (Fig. 3H). As expected, expression of the W92A mutant failed to restore U2AF1 protein levels, viral E protein level, and viral titers (Fig. 3I and J). These observations demonstrated an importance of U2AF1–U2AF2 heterodimerization in U2AF1 stability and viral replication.

### U2AF1 and U2AF2 facilitate the ZIKV protein synthesis

**Figure 4.**
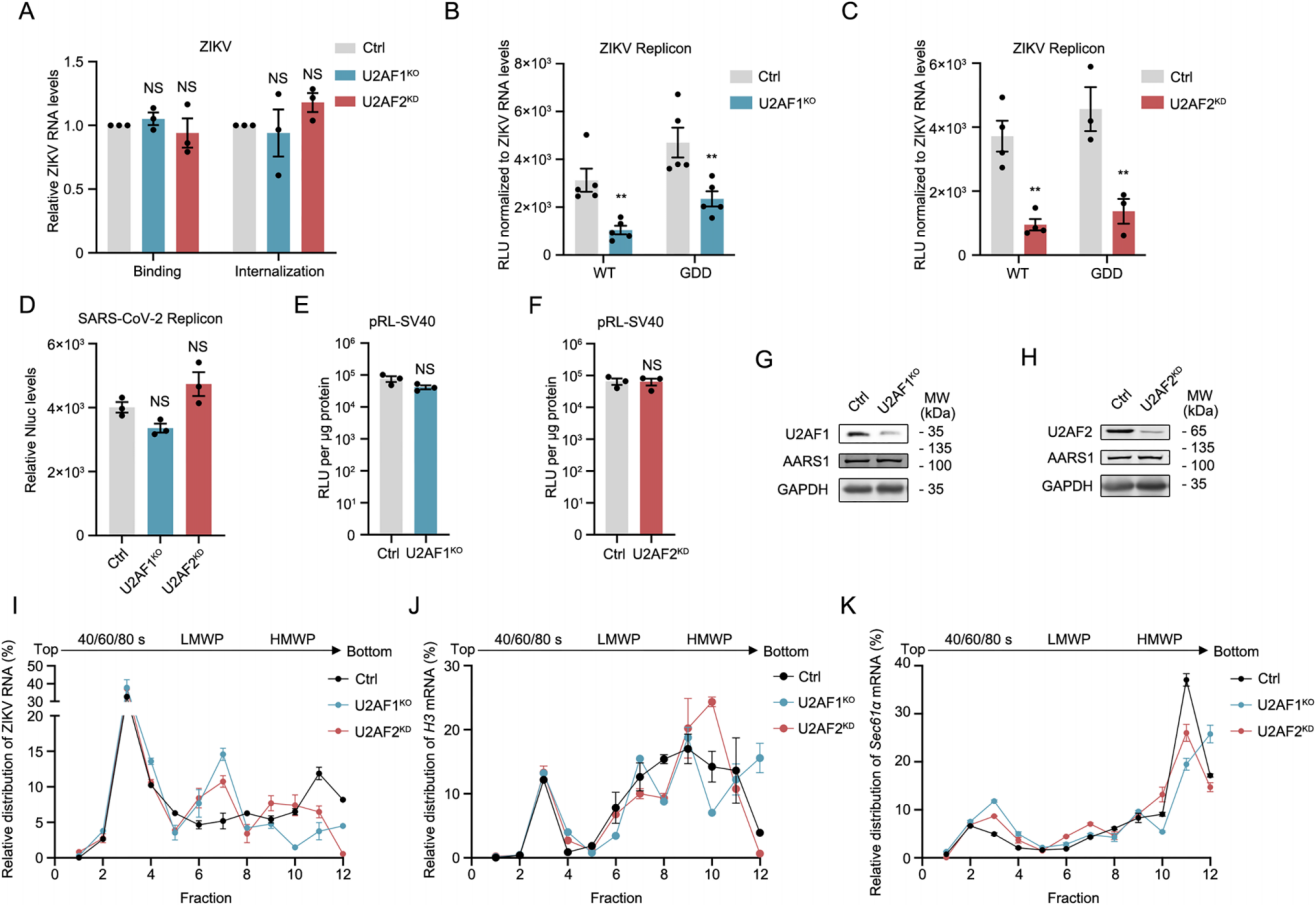
U2AF1 and U2AF2 promote viral protein synthesis. (**A**) Viral entry assay. Control, U2AF1^KO^, and U2AF2^KD^ cells were inoculated with ZIKV (MOI 3), followed by incubation either at 37°C for 1 h (virion binding) or at 37°C for 1 h (virion internalization). Cells were harvested for RT-qPCR. The data are shown as mean ± SEM from three biologically independent experiments. Statistical significance was determined using two-way ANOVA (NS, *P*  > .05, not significant). (**B–G**) Protein synthesis assay. Control, U2AF1^KO^, and U2AF2^KD^ cells were transfected with ZIKV WT or GDD replicon RNAs (**B, C**), SARS-CoV-2 replicon RNA (**D**), or pRL-SV40 reporter plasmid (**E, F**). Cells were harvested at 6 h p.t. for luciferase assay or for western blot (**G, H**). The data are shown as mean ± SEM from three biologically independent experiments. Statistical significance was determined using two-way ANOVA, one-way ANOVA, or two-tailed unpaired *t*-test, respectively (**P < .01 and NS, not significant). (I–K) Polysome profiling assay. Whole cell extracts of ZIKV-infected control, U2AF1^KO^, and U2AF2^KD^ were fractionated into 12 gradient fractions by sucrose gradient centrifugation. RNAs were isolated from each fraction and applied for RT-qPCR to detect the level of ZIKV RNA (**I**), *H3*, and *Sec61α* mRNA (**J, K**). LMWP, low molecular weight (mass) polysomes; HMWP: high molecular weight (mass) polysomes. Data are shown as mean ± SEM from three biologically independent experiments.

To delineate the molecular mechanism underlying U2AFs proviral function, we first determined the specific stage of viral replication they act at. To examine role of U2AF1/2 in the ZIKV attachment step, control, U2AF1^KO^, and U2AF2^KD^ cells were incubated with ZIKV virions for 1 h at 4°C. Cells were harvested for RNA extraction and RT-qPCR. The data revealed that the amounts of attached ZIKV virions were comparable in all tested cells (Fig. 4A). Then, we tested role of U2AF1/2 in viral internalization step. Cells were incubated with ZIKV virions, followed by incubation at 37°C for 1 h. The RT-qPCR data revealed comparable levels of internalized virions irrespective of U2AF1 and U2AF2 expression levels (Fig. 4A), indicating that U2AF1/2 do not regulate the viral entry step.

To examine whether U2AF1/2 acts at viral protein synthesis or RNA replication step, we employed wild-type (WT) and NS5^GDD^ mutant ZIKV replicons. The WT replicon encodes all seven nonstructural (NS) proteins while replacing the structural proteins with a Renilla luciferase reporter. The NS5^GDD^ replicon contains an inactivating mutation in the NS5 RNA-dependent RNA polymerase motif, rendering it replication-incompetent while maintaining translation capability [32]. Control, U2AF1^KO^, and U2AF2^KD^ cells were transfected with either WT or NS5^GDD^ replicon RNA, and harvested at 6 h p.t. for luciferase assay. We did not harvest cells at later time points because A549 cells were refractory to RNA transfection and died at 24 h p.t.. At 3 and 6 h p.t., the ZIKV RNA levels were comparable across all WT and NS5^GDD^ replicon RNA-transfected cells, indicating that depletion of U2AF1 or U2AF2 did not influence transfection efficiency or viral RNA stability (Supplementary Fig. S5A–D). The luciferase activities of WT and NS5^GDD^ ZIKV replicons in both U2AF1^KO^ and U2AF2^KD^ cells were significantly lower than those in the control cells (Fig. 4B and C), indicating that U2AF1 and U2AF2 regulate the protein synthesis of ZIKV. As a control, reduction of U2AF1 or U2AF2 did not affect the luciferase activities of a SARS-CoV-2 replicon and a pRL-SV40 reporter plasmid, nor did it alter the expression levels of endogenous proteins AARS1 and GAPDH (Fig. 4D–H).

To further evaluate the association of ZIKV RNA with translating ribosomes, we performed polysome fractionation assay. Whole cell lysates were separated by sucrose density gradient centrifugation, followed by RT-qPCR analysis to quantify ZIKV RNA levels in each ribosome fraction. In control cells, ZIKV RNA was predominantly associated with monosome fractions (fractions 2–4) and actively translating heavy polysome fractions (fractions 11–12) (Fig. 4I and Supplementary Fig. S5E). In contrast, in U2AF1^KO^ or U2AF2^KD^ cells, the relative distribution of ZIKV RNA shifted from heavy polysome fractions (11–12) toward light polysome fractions (6–8) (Fig. 4I). Notably, depletion of either U2AF1 or U2AF2 exerted no significant impact on the distribution patterns of two cytoplasm-translated mRNAs (*H3* and *ACTB*; Fig. 4J, and Supplementary Fig. S5F) and ER-translated mRNAs (*Sec61α* and *BiP*; Fig. 4K and Supplementary Fig. S5G). These results suggest that U2AF1 and U2AF2 might enhance viral protein synthesis by facilitating the association of viral RNA with heavy polysomes.

### U2AF1 and a truncated form of U2AF2 are located in the cytoplasm to support viral infection

**Figure 5.**
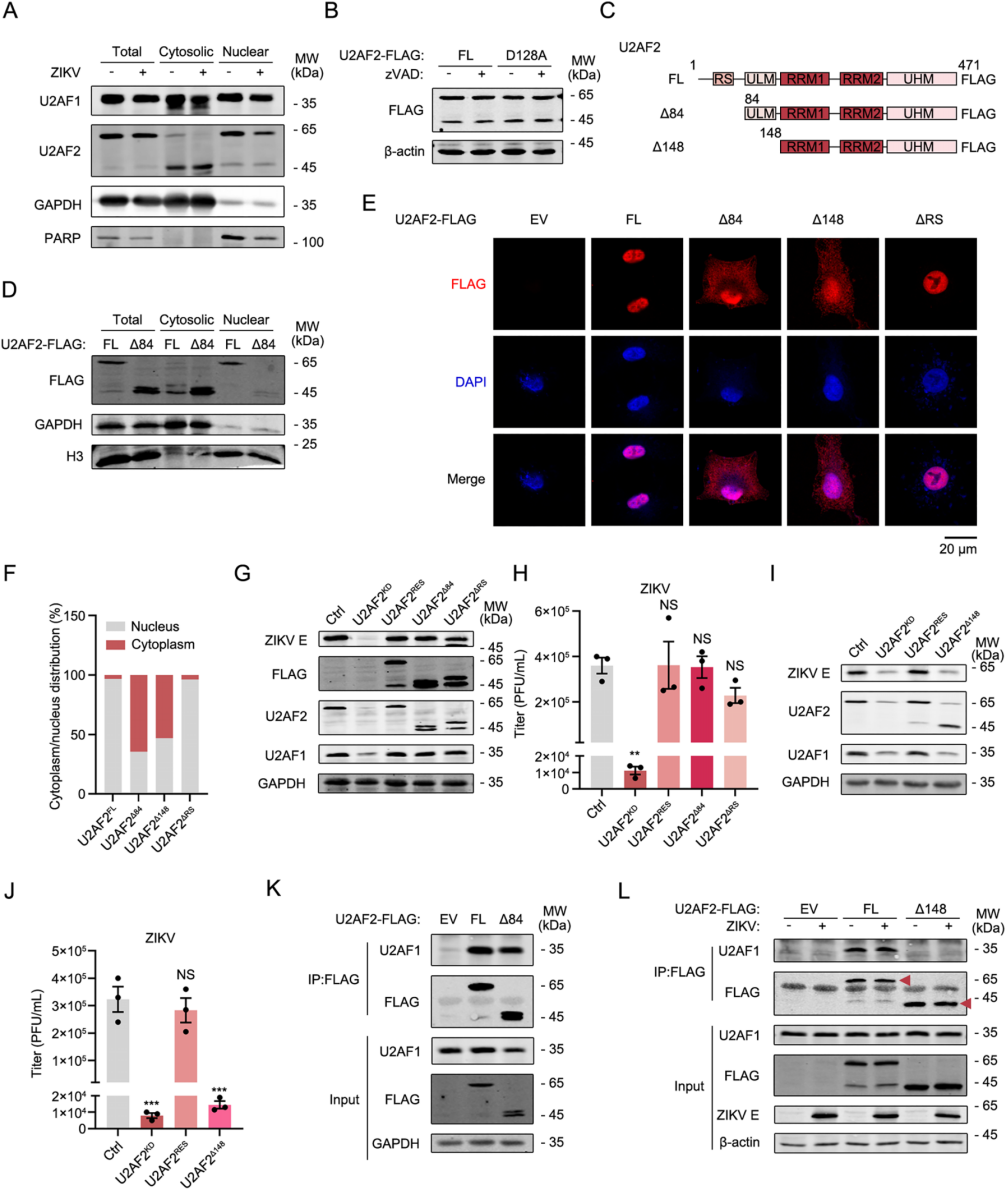
Functional characteristics of the U2AF2 45 kDa truncated forms. (**A**) Subcellular localizations of U2AF1 and U2AF2. A549 cells were infected with mock or ZIKV, and harvested 24 h p.i. for protein preparation. Proteins derived from cytoplasmic and nuclear fractions were subjected to western blot. GAPDH and PARP served as the loading control for cytoplasmic and nuclear fractions. (**B**) Impact of apoptosis inhibition on U2AF2 cleavage. Control and U2AF2^D128A^ cells were treated with DMSO or zVAD for 24 h and harvested for western blot using anti-FLAG and β-actin antibodies. (**C**) Schematic representation of FL U2AF2 and truncated mutants, U2AF2^Δ84^ and U2AF2^Δ148^. (**D**) Subcellular localization assay. U2AF2^FL^ and U2AF2^Δ84^ cells were harvested for protein extraction. Proteins derived from cytoplasmic and nuclear fractions were subjected to western blot. GAPDH and H3 served as the loading control for cytoplasmic and nuclear fractions. Representative images of three independent experiments are shown. (**E, F**) Immunofluorescence microscopy assay. U2AF2^RES^, U2AF2^Δ84^, U2AF2^Δ148^, and U2AF2^ΔRS^ cells were harvested for immunostaining using anti-Flag antibodies. DAPI stains the nuclei. Cell images were captured by Nikon C2 Confocal Microscope (Scale bars, 20 μm). The cytoplasmic and nuclear staining signals were quantified by Image J (F). Representative images of three independent experiments are shown. (**G**–**J**) Detection of viral replication levels. Control, U2AF2^KD^, U2AF2^RES^, U2AF2^ΔRS^, U2AF2^Δ84^, and U2AF2^Δ148^ cells were infected with ZIKV at an MOI of 3. At 24 h p.i., cells and supernatants were harvested for western blot and plaque assay. Statistical significance was determined using one-way ANOVA with Dunnett’s multiple comparison test (**P < .01, ***P < .001, and NS, not significant). Data are shown as mean ± SEM from three biologically independent experiments. (**K, L**) Co-IP assay. Whole cell extracts from cells expressing empty vector (EV), U2AF2^FL^, and U2AF2^Δ84^ were immunoprecipitated using anti-Flag antibody or control IgG. Samples were detected by western blot using anti-Flag, anti-U2AF1, and anti-GAPDH antibodies (**K**). Cells expressing empty vector (EV), U2AF2^FL^, and U2AF2^Δ148^ were infected with mock or ZIKV and harvested at 24 h p.i.. Whole-cell lysates were subjected to immunoprecipitation using anti-Flag antibody or control IgG. Samples were detected by western blot using anti-Flag, anti-U2AF1, anti-ZIKV E, and anti-GAPDH antibodies (**L**). Representative images of three independent experiments are shown.

As U2AF1 and U2AF2 are involved in the viral protein synthesis which occurs at cytoplasm, we further analyzed their subcellular distributions. A549 cells were infected with mock or ZIKV, and collected at 24 h p.i. for whole-cell extraction and subcellular fractionation. U2AF1 and U2AF2 were detected in both cytoplasmic and nuclear fractions, and their distribution patterns were similar between mock- and ZIKV-infected cells (Fig. 5A). Strikingly, two distinct forms of U2AF2 were observed in different cellular fractions: a 65-kDa FL form predominantly in the nuclear fraction, and a 45-kDa truncated form detected in the cytoplasmic fraction (Fig. 5A).

As previous study reported that U2AF2 undergoes caspase-mediated cleavage at aspartate 128 (D128) [40], we examined whether the observed 45 kDa truncated form of U2AF2 resulted from caspase-mediated cleavage in our model. First, we constructed a U2AF2^D128A^ mutant expression plasmid containing a D128A substitution, and transfected it into 293T cells. Surprisingly, the U2AF2^D128A^ mutant still expressed both the FL (65 kDa) and truncated (45 kDa) forms, identical to wild-type U2AF2 (Supplementary Fig. S5A). Consistently, pharmacological inhibition of caspases using z-VAD(OMe)-FMK (zVAD) did not reduce the level of 45 kDa truncated form in cells expressing either wild-type U2AF2 (U2AF2^FL^) or the U2AF2^D128A^ mutant (Fig. 5B). These data demonstrated that the generation of the 45 kDa U2AF2 form occurs through a caspase-independent mechanism.

The persistent appearance of the 45 kDa fragment in the U2AF2^ΔRS^ and U2AF2^ΔULM^ expressing cells (Fig. 3D) suggested that the cleavage site likely resides within either the 64–84 or 141–148 amino acid regions. To test this, we constructed two plasmids expressing truncated mutants of U2AF2: U2AF2^Δ64–84^ and U2AF2^Δ141–148^ (Supplementary Fig. S6B), and transfected them into 293T cells. Surprisingly, the 45 kDa cytoplasmic form of U2AF2 was still detected in the U2AF2^Δ64–84^ and U2AF2^Δ141–148^ expressing cells (Supplementary Fig. S6C), implying that there might be more than one protease cleavage sites present in the U2AF2 protein.

Given the cytoplasmic U2AF2 is mainly in a 45 kDa truncated form, we explored that whether this shorter fragment is sufficient to support ZIKV infection. We constructed two plasmids expressing truncated forms of U2AF2 approximating the size of the 45 kDa fragment: U2AF2^Δ84^ lacking 1–84 amino acids and U2AF2^Δ148^ lacking 1–148 amino acids (Fig. 5C). The sizes of U2AF2^Δ84^ and U2AF2^Δ148^ were close to the 45 kDa fragment of U2AF2 (Supplementary Fig. S6D), and both truncated proteins were partially localized in the cytoplasm as revealed by fractionation assay and immunofluorescence microscopy (Fig. 5D–F and Supplementary Fig. S6E–G). To examine whether that these cytoplasmic forms of U2AF2 are sufficient for its proviral effect, we measured the viral replication levels in the cells expressing endogenous U2AF2 (control), low level U2AF2 (U2AF2^KD^), ectopic U2AF2 (U2AF2^RES^), cytoplasmic U2AF2 (U2AF2^Δ84^ and U2AF2^Δ148^), or U2AF2^ΔRS^. The viral E protein levels and titers were largely rescued in the cells expressing U2AF2^RES^, U2AF2^Δ84^, and U2AF2^ΔRS^ (Fig. 5G and H). In contrast, the expression of U2AF2^Δ148^ failed to restore the downregulated levels of viral E and U2AF1 protein caused by U2AF2 knockdown (Fig. 5I and J), indicating an importance of 84–148 aa region (mainly ULM domain) in stabilizing U2AF1 and promoting viral replication. In addition, we performed co-IP assay to assess the interaction of U2AF2^Δ84^ or U2AF2^Δ148^ with U2AF1. U2AF2^Δ84^ was associated with U2AF1 (Fig. 5K). In contrast, U2AF2^Δ148^ failed to bind U2AF1 in the mock- and ZIKV-infected cells, which is expected due to its lack of the ULM domain (Fig. 5L). The above data demonstrated that U2AF1 and 45 kDa cytoplasmic form of U2AF2 are sufficient for their proviral function.

Considering that U2AF2^Δ84^ was localized predominantly in the cytoplasm, and that RS domain (1–64 aa) of U2AF2 has been implicated to serve as a nuclear localization signal (NLS) [41], we further compared the subcellular distributions of three truncated forms of U2AF2: U2AF2^ΔRS^ (lacking 1–64 aa), U2AF2^Δ64–84^, and U2AF2^Δ84^. As expected, FL U2AF2 was primarily nuclear, while U2AF2^Δ84^ was mainly cytoplasmic. Interestingly, both U2AF2^ΔRS^ and U2AF2^Δ64–84^ were predominantly localized in the nucleus, suggesting the presence of more than one functional NLS within the N-terminal region (1–84 aa) of U2AF2 (Supplementary Fig. S6H and I). As expected, expression of U2AF2^Δ64–84^ and U2AF2^Δ84^, similar to U2AF2^ΔRS^, in the U2AF2^KD^ cells could restore the ZIKV titers (Supplementary Fig. S6J).

### U2AF1 binds to ZIKV RNA in a U2AF2-dependent manner

**Figure 6.**
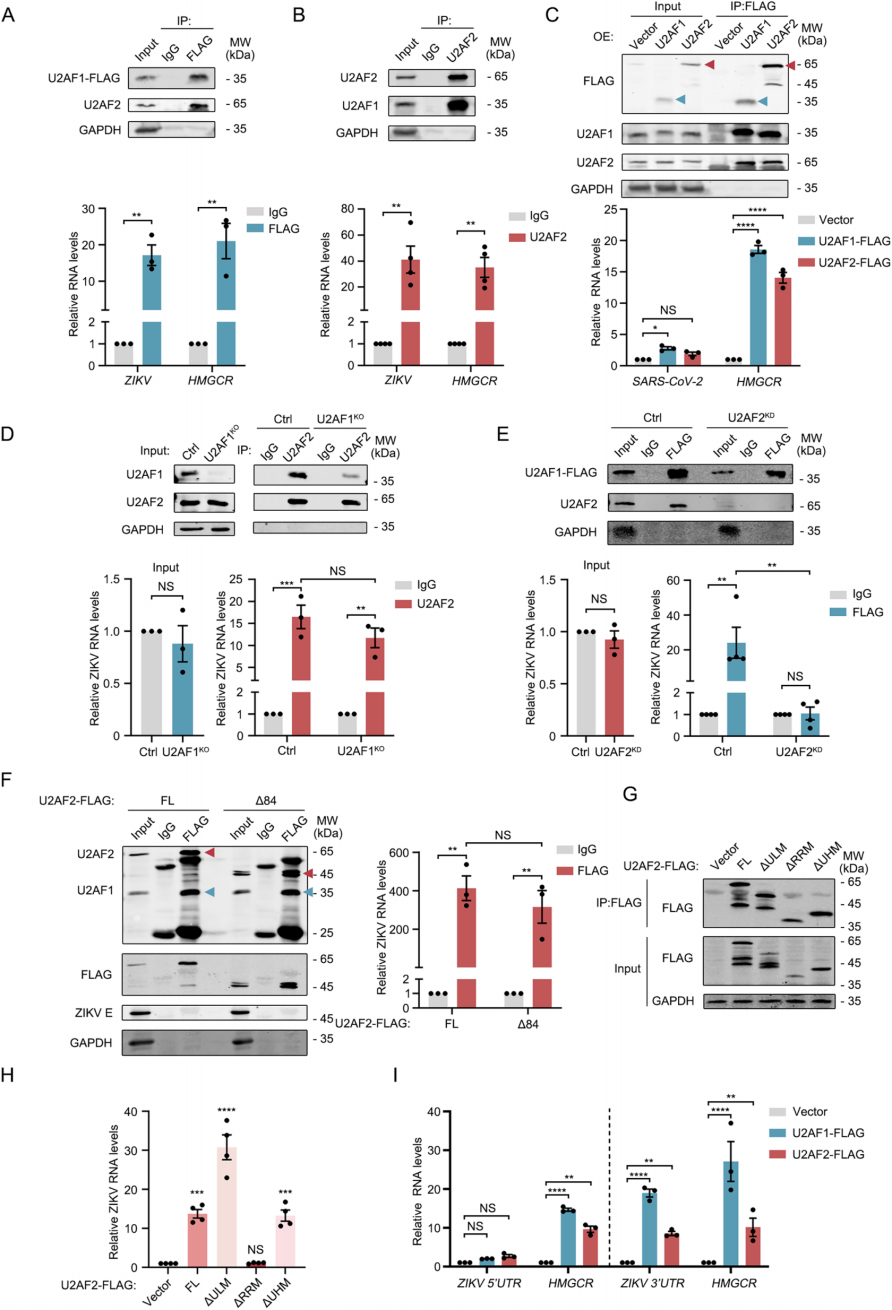
U2AF1 and U2AF2 bind to viral RNA. RIP assay was performed to detect the binding activity of U2AF1 and U2AF2 (full length or truncated forms) to ZIKV RNA. (**A**) Cells expressing U2AF1^RES^ were infected with ZIKV at an MOI of 3 for 24 h. (**B**) The ZIKV-infected A549 cells were harvested at 24 h p.i. (**C**) Control, U2AF1^RES^, and U2AF2^RES^ cells were transfected with SARS-CoV-2 replicon RNA and harvested at 6 h p.i. Control, U2AF1^KO^ cells (**D**), U2AF2^KD^ cells (**E**), U2AF2^FL^, and U2AF2^Δ84^ cells (**F**), or U2AF2 truncated mutants (ΔULM, ΔRRM, ΔUHM) (**G, H**) were transfected with ZIKV GDD replicon RNA for 6 h. (**I**) Control, U2AF1^RES^, and U2AF2^RES^ cells were transfected with 5′UTR or 3′UTR RNA of ZIKV and harvested at 6 h p.i. Whole cell extracts were prepared for RIP assay using either anti-Flag or anti-U2AF2 antibody. Samples were subjected to western blot using a mixture of anti-U2AF1 and anti-U2AF2 antibodies [panel (F)] or anti-FLAG antibody [panel (G)]. Total RNA in the precipitates was extracted for RT-qPCR to measure the ZIKV, SARS-CoV-2, and *HMGCR* RNA levels. Data are shown as mean ± SEM from three biologically independent experiments. Statistical significance was determined using two-way ANOVA or one-way ANOVA with Dunnett’s multiple comparison test (*P < .05, **P < .01, ***P < .001, ****P < .0001, and NS, not significant). Statistical significance of input RNA levels was determined using two-tailed unpaired *t*-test (NS, not significant).

As both U2AF1 and U2AF2 proteins contain RNA-binding domains and form a heterodimer, their contributions to bind viral RNA remain unclear. In order to explore their RNA-binding activity, we performed a series of RIP assays. First, control and U2AF1-overexpressing A549 cells were infected with mock or ZIKV, and harvested at 24 h p.i. for RIP assay. Both U2AF1 and U2AF2 were associated with ZIKV RNA and a positive control *HMGCR* mRNA [42] (Fig. 6A and B), but not the SARS-CoV-2 replicon RNA (Fig. 6C), confirming that they are specifically associated with ZIKV RNA. To determine which protein, U2AF1 or U2AF2, directly binds to ZIKV RNA, we compared the association of U2AF2 and viral RNA in control and U2AF1^KO^ cells. To ensure equivalent input of viral RNA, equal amounts of NS5^GDD^ mutant replicon RNA were transfected into control and U2AF1^KO^ cells. The RT-qPCR data confirmed that the input ZIKV RNA levels were comparable in control and U2AF1^KO^ cells (Fig. 6D). The amounts of ZIKV RNA binding U2AF2 were similar regardless of the absence and presence of U2AF1 (Fig. 6D), indicating that U2AF2 binds viral RNA independently of U2AF1. Next, we assessed the association of U2AF1 with ZIKV RNA in the U2AF2 sufficient (control) and deficient (U2AF2^KD^) cells. In the U2AF2^KD^ cells, the amount of viral RNA binding U2AF1 was dramatically reduced compared to the control cells (Fig. 6E). These RIP assay data revealed that U2AF2, rather than U2AF1, directly binds to ZIKV RNA.

To assess whether the cytoplasmic U2AF2 truncated form can bind to ZIKV RNA, we compared the viral RNA levels associated with FL (U2AF2^FL^) and cytoplasmic truncated form U2AF2 (U2AF2^Δ84^). The data revealed that U2AF2^Δ84^ binds to ZIKV RNA with an affinity comparable to that of U2AF2^FL^ (Fig. 6F), demonstrating that the cytoplasmic form of U2AF2 largely retains the ability to bind viral RNA. Next, we mapped the domain responsible for ZIKV RNA binding within U2AF2^Δ84^, which comprises the ULM, RRM, and UHM regions. As shown in Fig. 6H, deletion of the RRM domain of U2AF2, but not other domains, abolished its binding to ZIKV RNA, indicating that the RRM domain is essential for flaviviral RNA interaction. Furthermore, to map the flaviviral RNA region involved in binding, we assessed the interaction between U2AF1/2 and the ZIKV 5′UTR and 3′UTR. U2AF1/2 exhibited high binding affinity specifically for the ZIKV 3′UTR, but not the 5′UTR (Fig. 6I). These data suggested that the 3′UTR of the ZIKV genome interacts with U2AF heterodimers, and potential binding between the CDS and U2AF cannot be excluded.

### Mosquito u2af heterodimers facilitate *in vivo* ZIKV infection in *A. aegypti*

**Figure 7.**
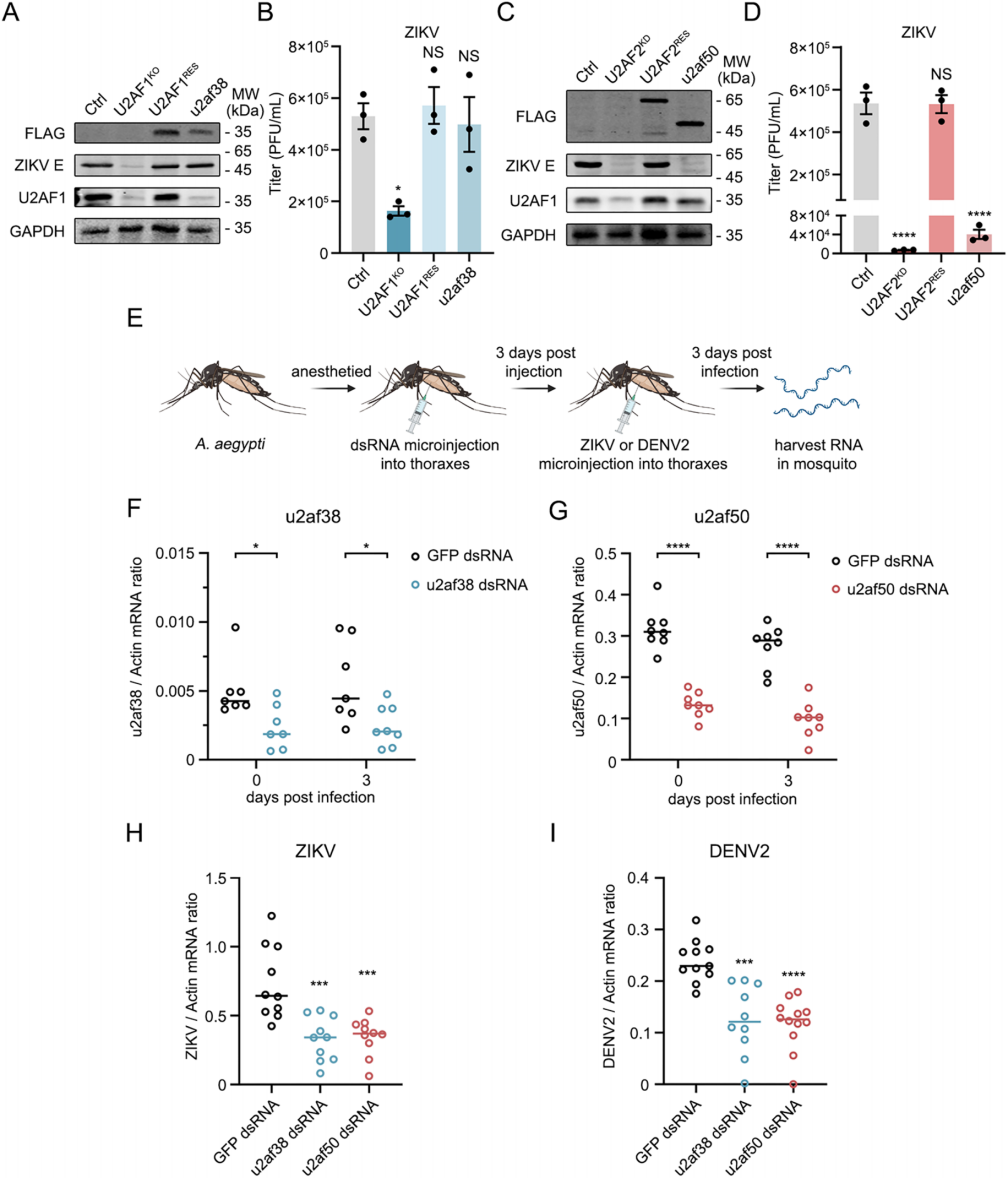
Role of mosquito u2af38 and u2af50 in ZIKV replication. (A–D) Roles of mosquito u2af38 and u2af50 in the ZIKV infection in human cells. Control, U2AF1^KO^, U2AF1^RES^, and U2AF1^KO^ expressing u2af38 cells (**A, B**), or control, U2AF2^KD^, U2AF2^RES^, and U2AF2^KD^ expressing u2af50 cells (**C, D**) were infected with ZIKV. At 24 h p.i., cells and supernatants were harvested for western blot and plaque assay. Representative images of three independent experiments are shown. Data are shown as mean ± SEM from three biologically independent experiments. Statistical significance was determined using one-way ANOVA with Dunnett’s multiple comparison test (*P < .05, ****P < .0001, and NS, not significant). (**E**) A schematic representation of the *in vivo* study design. Created in BioRender (Huang C., 2026, https://BioRender.com/hgx1mgg). (F–I) RT-qPCR assay. *Aedes aegypti* mosquitoes were microinjected with dsRNAs targeting u2af38, u2af50, or GFP as a negative control. The levels of u2af38 (**F**), u2af50 (**G**), or viral RNA (**H, I**) were measured by RT-qPCR and normalized against *A. aegypti actin*. Each dot represents one mosquito. The horizontal lines indicate the median. Statistical significance was determined using two-way ANOVA or one-way ANOVA with Dunnett’s multiple comparison test (*P < .05, ***P < .001, and ****P < .0001).

Given that mosquito-borne flaviviruses cycle between vertebrate hosts and insect vectors, we examined whether mosquito U2AF homologs (u2af38 and u2af50) play a similar role in viral replication in human cells and *in vivo* mosquitoes. Sequence alignment revealed that mosquito u2af38 and u2af50 share 75.49% and 63.79% amino acid identity with human U2AF1 and U2AF2, respectively (Supplementary Fig. S7A and B). We first tested whether *trans*-complementation with mosquito u2af38 or u2af50 in the U2AF1^KO^ or U2AF2^KD^ cells could restore ZIKV replication. Expression of u2af38 in the U2AF1^KO^ cells substantially rescued both viral E protein levels and ZIKV titers (Fig. 7A and B). Surprisingly, although expression of u2af50 in the U2AF2^KD^ cells increased U2AF1 protein levels, it did not restore ZIKV replication (Fig. 7C and D). These results indicate that mosquito u2af38, but not u2af50, can functionally substitute for its human homolog in supporting viral infection.

Then, we examined whether loss of mosquito u2af38 and u2af50 has an impact on the *in vivo* infection of ZIKV using dsRNA-mediated gene silencing in *A. aegypti*. Female *A. aegypti* mosquitoes were microinjected with dsRNAs targeting either control (GFP), u2af38, or u2af50, and then inoculated with ZIKV or DENV2. At 3 days p.i., individual mosquitoes were collected for RNA extraction and RT-qPCR analysis (Fig. 7E). The mRNA levels of u2af38 and u2af50 were significantly reduced in mosquitoes treated with the corresponding dsRNAs (Fig. 7F and G), confirming efficient knockdown. Notably, viral loads of both ZIKV and DENV2 were significantly reduced in the u2af38- and u2af50-silenced mosquitoes compared to the control group (Fig. 7H and I), indicating that these mosquito U2AF homologs are also essential for flavivirus infection in mosquitoes.

## Discussion

During the life cycle, flaviviruses rely on the recruitment of numerous host factors to facilitate their replication. To systematically identify these factors, we conducted an unbiased screen using MS-based proteomic profiling of ER-enriched fractions, given that flavivirus biosynthesis primarily occurs at the ER. Combined with functional validations, our study established the essential roles of two splicing factors, U2AF1 and U2AF2, in both *in vitro* and *in vivo* viral infection. Several key findings emerged from this work.

First, we demonstrated that both U2AF1 and U2AF2 are required for flavivirus replication in multiple cell lines. As complete knockout of U2AF2 proved lethal [43], we utilized multiple approaches: CRISPR-Cas9-mediated gene editing for U2AF1, and RNAi strategies (siRNA and shRNA) for both U2AF2 and U2AF1. Depletion of either U2AF1 or U2AF2 resulted in a significant reduction in ZIKV and DENV2 replication, which could be largely restored upon *trans*-complementation with exogenous U2AF1 or U2AF2 in the U2AFs-deficient cells. Interestingly, U2AF1 and U2AF2 do not regulate the replication of other RNA viruses such as coronavirus or alphavirus, suggesting that flaviviruses have evolved a distinct mechanism to exploit these host splicing factors.

Our study reveals that U2AF1 and U2AF2 facilitate viral protein synthesis, a finding that contrasts with a previous JEV study [30], which proposed that U2AF2 promotes viral RNA synthesis. It is well established that flaviviral RNA and protein levels are interdependent and can influence each other. Therefore, the conclusion drawn in the JEV study, which was based solely on the observation that U2AF2 knockdown reduced both viral RNA and NS5 protein levels, may not be accurate. This interpretation is further weakened by the fact that JEV RNA levels before 12 h p.i. were unaffected by U2AF2 silencing. In contrast, data from our ZIKV replicon and polysome fractionation assays provide more direct and compelling evidence supporting a role for U2AF2 and U2AF1 in viral protein synthesis. Of note, depletion of U2AF1 and U2AF2 suppressed the translation of GDD mutant replicon RNA, indicating that they modulate the initial round of replication-independent protein synthesis. Whether they participate in replication-dependent protein synthesis remains to be elucidated. U2AF1 and U2AF2 depletion did not affect the translation of a Renilla reporter and a SARS-CoV-replicon RNA, demonstrating that their role in protein synthesis is virus specific. Intriguingly, polysome fractionation assays revealed that U2AF2 knockdown slightly yet non-significantly reduced the abundance of host mRNAs within fractions 11–12, implicating a role of U2AF2 in the regulation of host mRNA translation [44, 45]. Multiple lines of evidence corroborate a functional role for the U2AF complex in protein synthesis: the nucleocytoplasmic shuttling of both U2AF1 and U2AF2 [20], the binding of U2AF35 to spliced mRNA [46], and the association of Drosophila U2AF50 with intronless mRNAs [47]. Collectively, these findings suggest that U2AF complexes promote translation of certain proteins.

Given that viral protein synthesis primarily occurs in the cytoplasm, we conducted a detailed investigation into the subcellular localizations of U2AF1 and U2AF2. In uninfected cells, both proteins were predominantly nuclear, and following ZIKV infection, no significant alteration in their localization patterns was observed (Fig. 5A). Notably, we identified a truncated form (45 kDa) of U2AF2 in the cytoplasm, which was proved to be sufficient to support viral replication. Since ectopic expression of FL U2AF2 also produced this 45 kDa fragment, we propose that it likely arises from internal ATGs or protease-mediated cleavage rather than alternative splicing. To identify potential internal ATGs or protease cleavage site(s) within U2AF2, we analyzed the expression patterns of a series of truncated mutants: (i) expression of the U2AF2^D128A^ mutant yielded both 65 kDa and 45 kDa fragments, similar to wild-type U2AF2, indicating that residue 128D is not a caspase cleavage site. The discrepancy between our cellular data and the previous *in vitro* finding [40] could be explained by the lack of cellular regulation in purified systems. Purified caspases can exhibit off-target activity and cleave sites that are not accessible or recognized in the intact cellular environment. (ii) Expression of four truncation mutants spanning the 1–148 aa region (ΔRS:1–64 aa; Δ64–84 aa; ΔULM:84–141 aa; Δ141–148 aa) also produced the 45 kDa cytoplasmic fragment (Fig. 3D and Supplementary Fig. S6C), suggesting the presence of multiple potential internal ATGs or protease cleavage sites within this region. Combined with the observations that Δ84 generates two fragments, while Δ148 expresses only one (Supplementary Fig. S5D), four potential internal ATG codons, including M105, M110, M125, and M144, were identified as possibly initiating translation of the 45 kDa fragment. The presence of these internal ATGs may also explain why FL U2AF2 and ΔRS produce a fragment larger than that of ΔULM (Δ84–141) (Fig. 3D). Specifically, translation initiation at M105, M110, or M125 yields the 45 kDa fragment, whereas initiation at M144 leads to the production of a smaller fragment. We excluded the possibility that U2AF2 is cleaved by the ZIKV protease NS2B3, as the 45 kDa fragment was also detected in the cytoplasmic fraction of mock-infected cells. The biological implications of cytoplasmic localization of truncated U2AF2 warrant further investigation. It should be noted that in the manuscript by Song *et al*. [30], the authors did not provide a full western blot image, making it difficult to assess the band pattern of U2AF2 protein. Surprisingly, their blots showed a significant downregulation of FL U2AF2 following JEV infection. Moreover, as flavivirus RNAs are synthesized within ER-derived ROs, their conclusion that nuclear U2AF2 is translocated to the cytoplasm through binding to JEV RNAs lacks experimental support [30].

Interestingly, we identified a novel NLS within the 64–84 aa region of U2AF2. Both U2AF2^ΔRS^ (lacking residues 1–64) and U2AF2^Δ64–84^ were efficiently transported into the nucleus, whereas U2AF2^Δ84^ was predominantly localized in the cytoplasm (Fig. 5D and Supplementary Fig. S6H). These findings indicate that both the 1–64 aa and 64–84 aa regions can function independently as NLSs to facilitate nuclear import of U2AF2 (Summarized in Supplementary Fig. S8). Consistent with this, sequence analysis revealed that both regions are enriched in basic amino acids (lysine and arginine) (Supplementary Fig. S7A and B), a feature of classical NLSs.

In addition to evaluating the contributions of individual domains of U2AF2 to its subcellular localization, we also determined the roles of these domains in the proviral functions of U2AF1 and U2AF2. For U2AF1, the Zn1 and RS domains were essential, whereas the Zn2 domain was dispensable; the role of the RRM domains remains unclear. In U2AF2, the ULM, RRM, and UHM domains were all functionally involved, while the RS domain was dispensable. As expected, we demonstrated that the RRMs of U2AF2 are responsible for binding ZIKV RNA (Fig. 6H). Notably, we were unable to detect expression of the U2AF1^ΔRRM^ mutant, likely because U2AF1 requires heterodimerization with U2AF2 for stability through RRM-binding to the ULM domain of U2AF2. This hypothesis is supported by the reduced accumulation of U2AF1 protein in both U2AF2^KD^ and U2AF2^ΔULM^ cells, but not in cells expressing other truncated forms of U2AF2 (Fig. 3D). Treatment of U2AF2^KD^ cells with MG132 (a proteasome inhibitor) or NH_4_Cl (a lysosomal degradation inhibitor) failed to restore U2AF1 accumulation (data not shown), suggesting that U2AF1 degradation occurs independently of these pathways. The mechanism underlying U2AF2-mediated stabilization of U2AF1 requires further investigation.

The functional significance of the U2AF1–U2AF2 interaction in viral replication was further corroborated by inhibitor assay. Treatment with 7,8-dihydroxyperphenazine significantly suppressed flavivirus infection. It should be noted, however, that 7,8-dihydroxyperphenazine may also disrupt interactions of other proteins containing UHM/ULM domains—such as splicing factor 45 (SPF45), RNA-binding protein 39 (RBM39), and poly(U)-binding-splicing factor PUF60 [48, 49]. Therefore, the antiviral effect of 7,8-dihydroxyperphenazine cannot be solely attributed to inhibition of the U2AF1/U2AF2 interaction. Nonetheless, the W92A point mutation in U2AF2, which abolishes the interaction of U2AF1 and U2AF2, failed to rescue the viral replication in the U2AF2^KD^ cells (Fig. 3G–J), strengthening the importance of U2AF1–U2AF2 interaction in supporting viral replication.

We conclude that the proviral roles of U2AF1 and U2AF2 are non-redundant, based on the following observations: (i) knockout of U2AF1 reduced viral replication without affecting U2AF2 expression (Fig. 2C and D); (ii) although expression of U2AF2^ΔRRM^, U2AF2^ΔUHM^, or mosquito u2af50 in the U2AF2^KD^ cells restored the U2AF1 protein accumulation, the viral replication was not correspondingly rescued (Figs 3D and F, and 7C and D), indicating that U2AF1 cannot functionally compensate for the loss of U2AF2 during viral infection. Instead, U2AF1 and U2AF2 function synergistically to enhance viral protein synthesis. U2AF2 binds directly to viral RNA via its RRM domain and concurrently stabilizes U2AF1 through protein–protein interactions. U2AF1 might control translation either through its Zn1 domain, which has been reported to increase translation when localized to the 5′UTR of a model mRNA [50], or through its RS domain in a manner analogous to other SR family proteins, such as SRp20, which supports poliovirus replication by linking the viral IRES to the host translation machinery [51, 52]. Nonetheless, the role of U2AF2 in flavivirus replication appears distinct from its functions in other viral infections. For instance, U2AF2 contributes to the proper processing of HIV transcripts and modulates RNA splicing in HPV and IAV infections [25, 26]. So far, U2AF1 and U2AF2 have been implicated in the replication of viruses that replicate in nuclei. In this study, we present compelling evidence that U2AF1 and U2AF2 are critical for the cytoplasmic replication of flaviviruses.

Finally, we demonstrated that the mosquito homologs of U2AF1 and U2AF2, u2af38 and u2af50, similarly promote flavivirus replication in *A. aegypti* (Fig. 7H and I). Sequence alignment analyses revealed that mosquito u2af38 and u2af50 contain all domains highly similar to those of their human counterparts (Supplementary Fig. S7A and B), consistent with their conserved role as essential splicing factors in pre-mRNA processing. Our findings demonstrate that expression of mosquito u2af38 in U2AF1^KO^ cells restores ZIKV replication. In contrast, mosquito u2af50 was unable to compensate for the loss of U2AF2 despite recovering U2AF1 protein levels, suggesting that while mosquito u2af50 retains certain conserved functions, such as stabilizing U2AF1, it cannot fully substitute for human U2AF2 in supporting viral replication. Further structural and functional comparisons between human and mosquito U2AF2 will be pursued in future studies. Importantly, in mosquitoes, the knockdown of either u2af38 or u2af50 reduced ZIKV replication *in vivo*, supporting the functional conservation of these splicing factors across species. Due to the lethality of constitutive U2AF2 knockout in mice, we were unable to assess the impact of murine U2AF1 and U2AF2 on viral infection and pathogenesis. Future studies could employ conditional knockout mouse models to address this question.

In summary, we propose a model for the proviral mechanism of U2AF1 and U2AF2 during flavivirus infection. In latent cells, U2AF1 and U2AF2 are predominantly nuclear, while a small fraction of U2AF1 and cleaved U2AF2 reside in the cytoplasm. Following flaviviral entry, these cytoplasmic U2AF1 and U2AF2 are recruited by viral genomic RNAs to the ER. There, U2AF1 and U2AF2 coordinate to promote viral protein synthesis by facilitating the association of flaviviral RNA with heavy polysomes. This study comprehensively characterizes the structural and functional features of U2AF1 and U2AF2 in flaviviral infection both *in vitro* and *in vivo*, and provides compelling evidence elucidating the molecular basis of their proviral roles. These findings underscore the potential of U2AF1 and U2AF2 as targets for the development of broad-spectrum antiviral therapies.

## Supplementary Material

gkag713_Supplemental_File

## Data Availability

Source data are provided with this paper. The mass spectrometry proteomics data generated in this study have been deposited via the ProteomeXchange Consortium in the iProX partner repository with the dataset identifier IPX0013687000. Source data are provided with this paper.
