# Supplementary material for: Exploitation of host U2AF1 and U2AF2 splicing factors facilitates mosquito-borne orthoflavivirus infection across species
Source: Nucleic Acids Res. 2026 Jul 17;54(14):gkag713. doi: 10.1093/nar/gkag713 (PMC13376149; doi:10.1093/nar/gkag713)
Supplement: gkag713_Supplemental_File [file gkag713_supplemental_file.pdf]

Supplemental figures

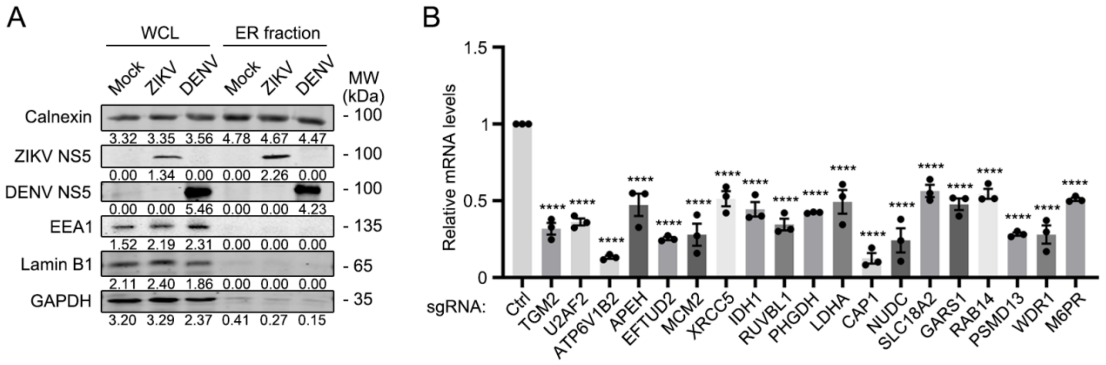

Fig. S1. Isolation and screen of ER-enriched proteins. (A) Examination of ER fraction purity. Huh7.5 cells were infected with mock or ZIKV. At 24 h p.t., cells were collected for ER extraction. Samples of ER fractions were analyzed by western blot using antibody against ER marker Calnexin, viral NS5 proteins, endosomal EEA1, nuclear Lamin B1, and cytoplasmic GAPDH. (B) Detection of gene-editing efficiency. Gene-edited Huh7.5 bulk cells were generated by lentivirus-mediated transduction and drug selection. Total RNAs were extracted for real-time PCR using their specific primers. The data are shown as mean ± SEM from three biologically independent experiments. Representative images of three independent experiments are shown. Statistical significance was determined using one-way ANOVA with Dunnett's multiple comparison test (****$p < 0.0001$).

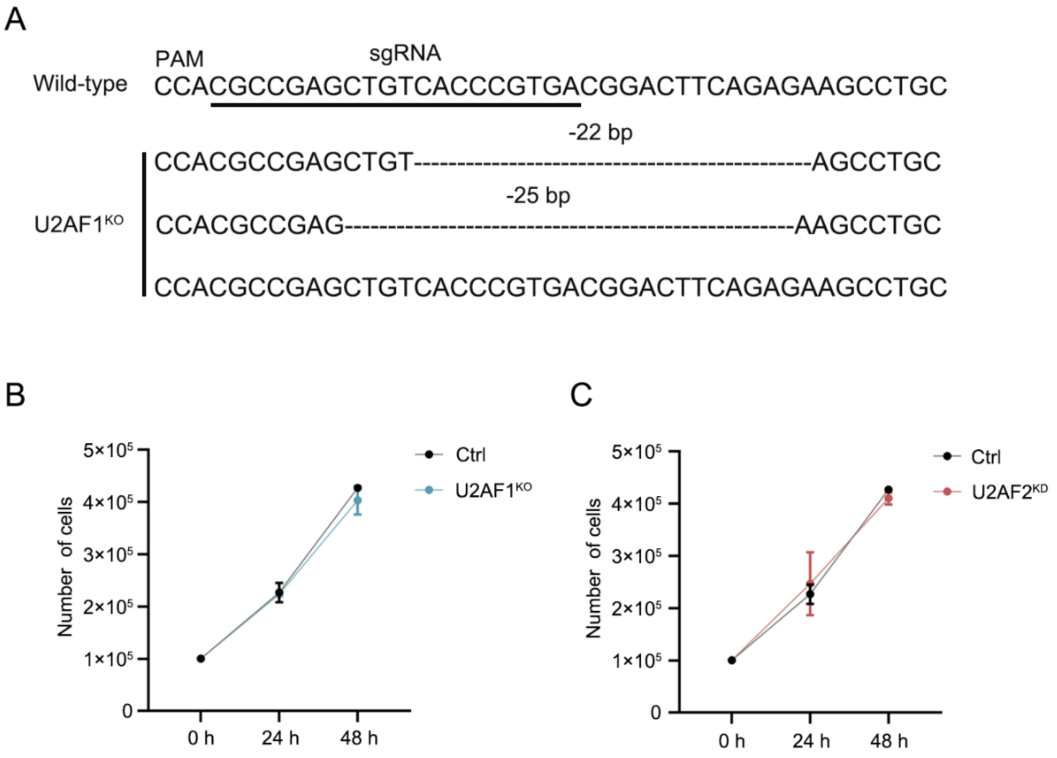

Fig. S2. Characteristics of U2AF1 and U2AF2 deficient cells. (A) Sequencing of U2AF1^{KO} cells. Genomic DNA was extracted from U2AF1^{KO} cells, and the region surrounding the sgRNA target site was PCR-amplified. The PCR products were then subjected to Nanopore sequencing. (B, C) Growth rates of U2AF1^{KO} and U2AF2^{KD} cells. Same amounts of cells (1×10^5 each well in 12-well plate) were seeded. At 1 and 2 days, cell were counted. The data are shown as mean ± SEM from three biologically independent experiments.

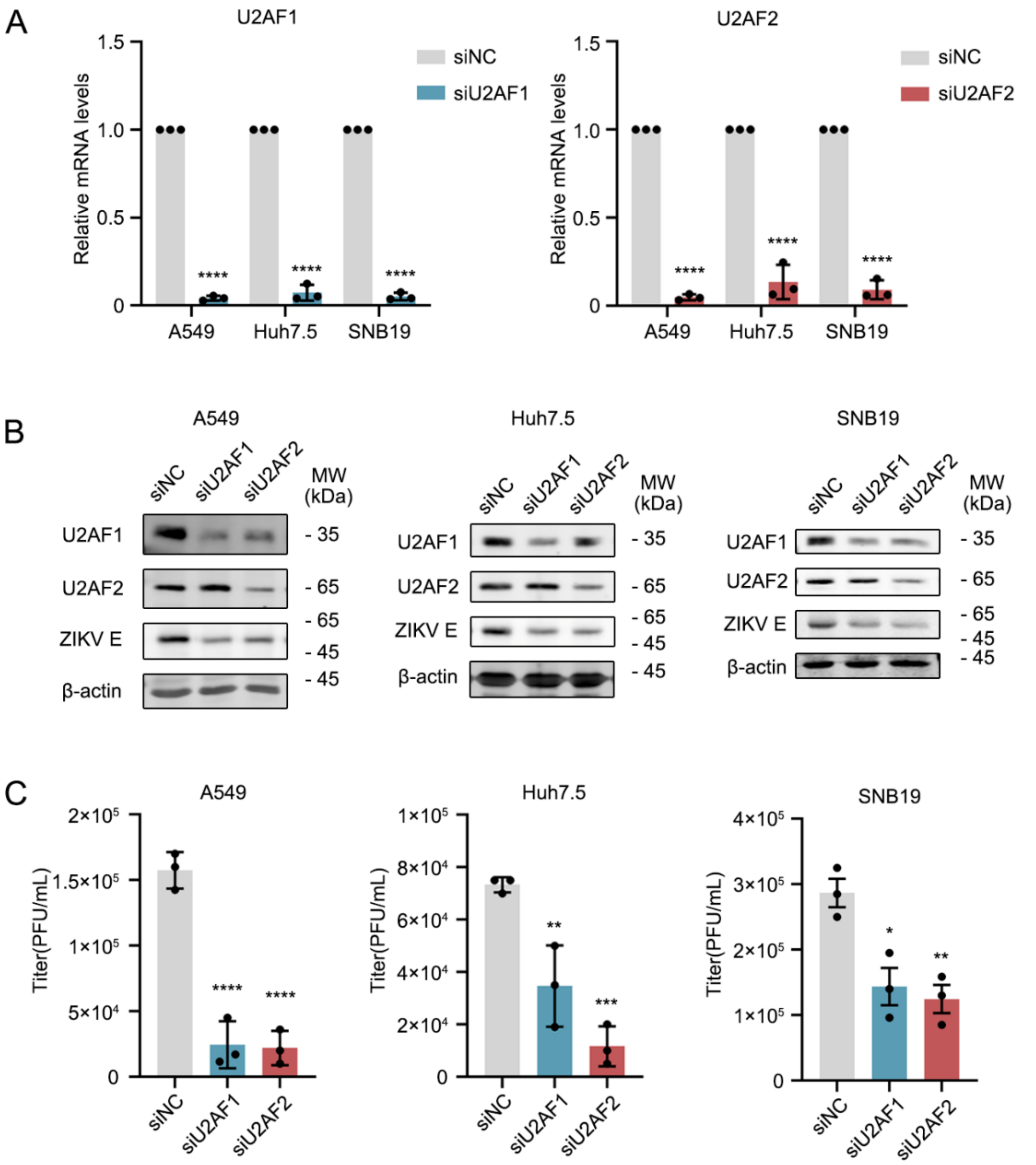

Fig. S3. U2AF1 and U2AF2 promote ZIKV infection in A549, Huh7.5, and SNB19 cells. (A) Efficiencies of siRNAs targeting U2AF1 or U2AF2. A549, Huh7.5, or SNB19 cells were transfected with siNC, siU2AF1 or siU2AF2. At 48 h p.t., cells were harvested for qRT-PCR. (B, C) Detection of viral replication levels. Cells were transfected with siNC, siU2AF1 or siU2AF2 for 48 h, followed by ZIKV infection. At 24 h p.i., cells and supernatants were harvested for western blot (B) and plaque assay (C). The data are shown as mean ± SEM from three biologically independent experiments. Representative images of three independent experiments are shown. Statistical significance was determined using one-way ANOVA with Dunnett's multiple comparison test (*p < 0.05, **p < 0.01, ***p < 0.001, and ****p < 0.0001).

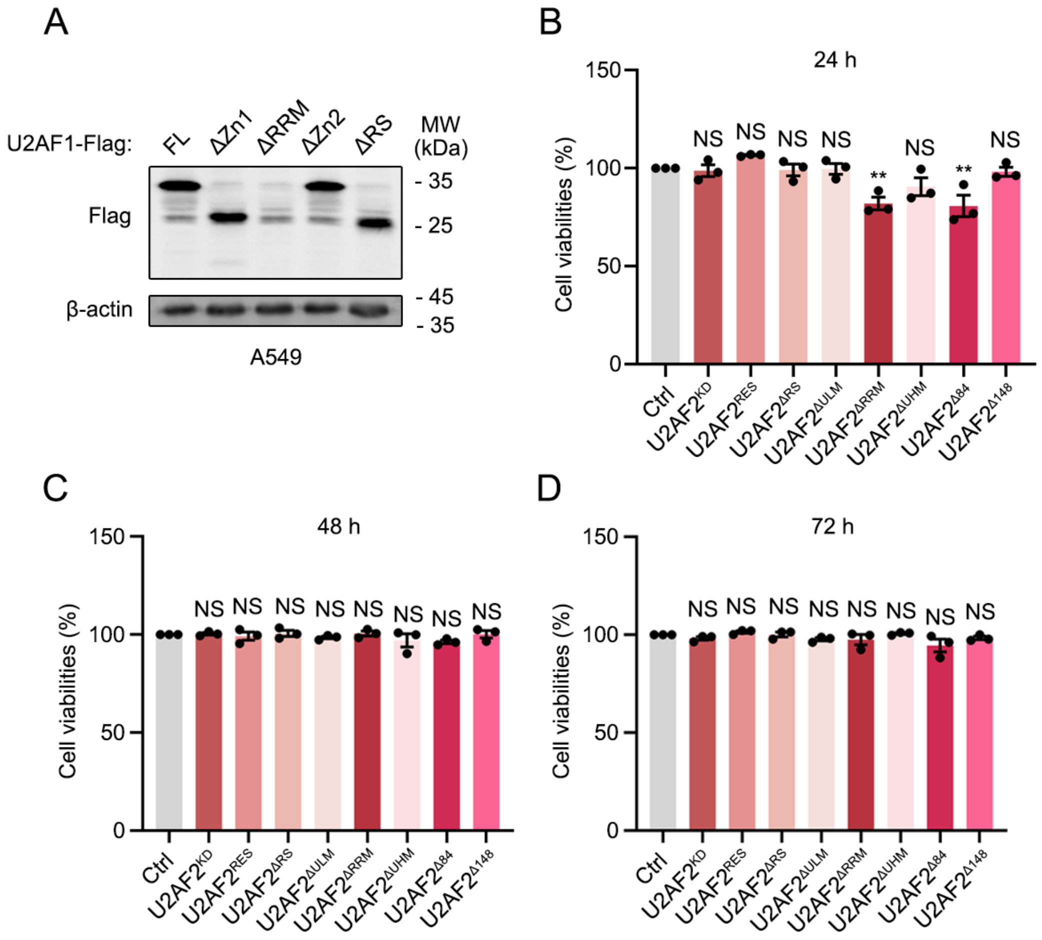

Fig. S4. Characteristics of U2AF1 truncated forms and cells expressing U2AF2 truncated forms. (A) Cells expressing U2AF1$^{\Delta Zn1}$, U2AF1$^{\Delta RRM}$, U2AF1$^{\Delta Zn2}$, or U2AF1$^{\Delta RS}$ were harvested for western blot using anti-Flag antibody. Representative images of three independent experiments are shown. (B-D) Cell viability assay. Control, U2AF2^{KD}, and U2AF2^{KD} expressing full-length U2AF2 (rescue, U2AF2^{RES}) or different truncated forms U2AF2 cells were seeded, and harvested at indicated time points (24 h, 48 h, or 72 h) for CCK8 assay. The data are shown as mean ± SEM from three biologically independent experiments. Statistical significance was determined using one-way ANOVA with Dunnett's multiple comparison test (**$p < 0.01$ and NS, not significant).

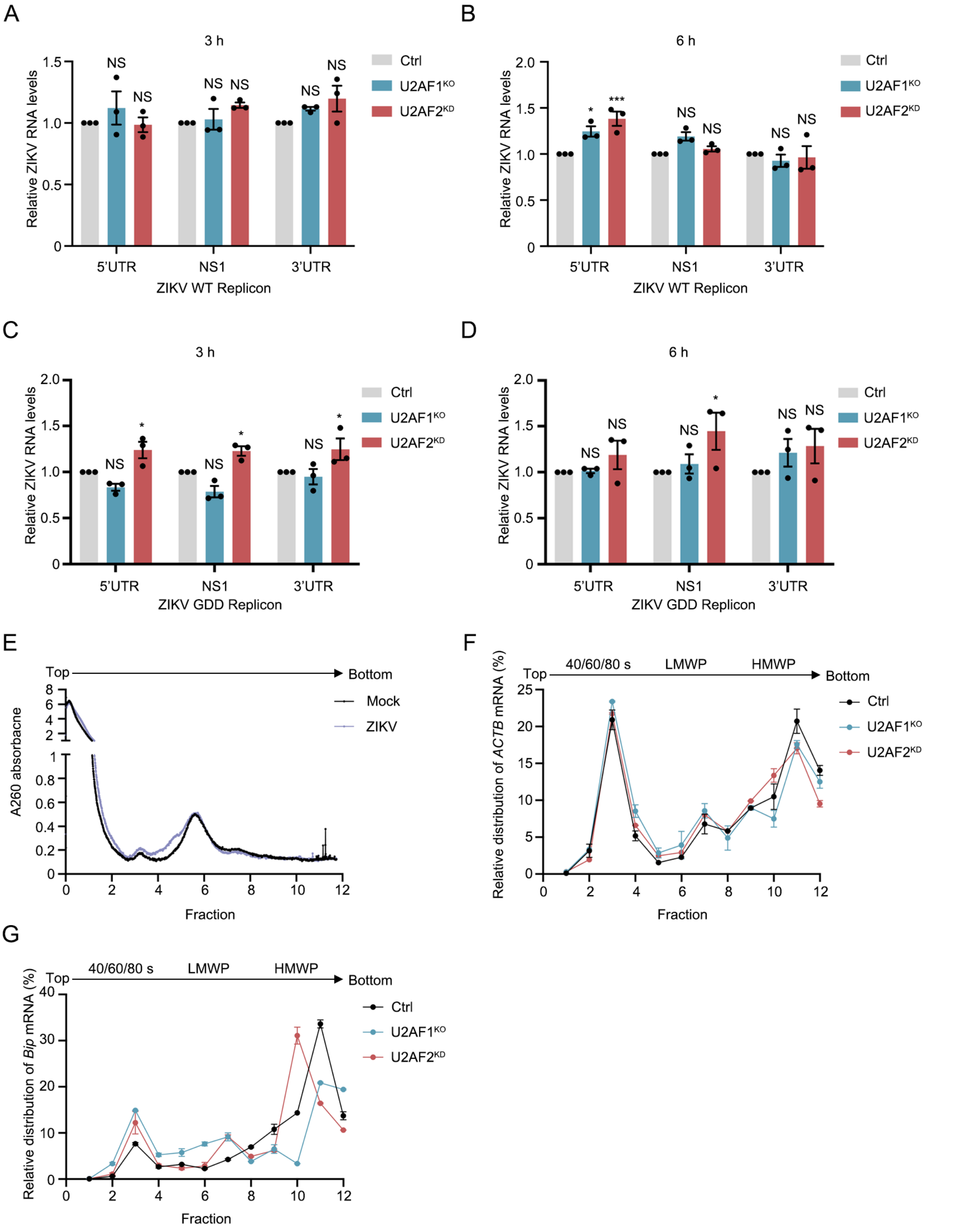

Fig. S5. Depletion of U2AF1 and U2AF2 did not affect ZIKV RNA stability or host mRNA translation efficiency. (A-D) RNA stability assay. Control, U2AF1^{KO}, and U2AF2^{KD} cells were transfected with ZIKV WT (A-B) or GDD (C-D) replicon RNAs and harvested at 3 h and 6 h p.i.. Total RNA was extracted for RT-qPCR to measure the *5'UTR*, *NS1*, and *3'UTR* RNA levels. Statistical significance was determined using two-way ANOVA with Dunnett's multiple comparison test (*p < 0.05, ***p < 0.001 and NS, not significant). Data are shown as mean ± SEM from three biologically independent experiments. (E) Polysome profiling assay. Whole cell extracts of mock and ZIKV-infected cells were fractionated into 12 gradient fractions by sucrose gradient

centrifugation. A260 absorbance of each fraction was detected. (F, G) Polysome profiling assay. Whole cell extracts of ZIKV-infected control, U2AF1^{KO}, and U2AF2^{KD} were fractionated into 12 gradient fractions by sucrose gradient centrifugation. RNAs were isolated from each fraction and applied for RT-qPCR to detect the level of *ACTB* (F) and *Bip* (G) mRNA. LMWP, low molecular weight (mass) polysomes; HMWP: high molecular weight (mass) polysomes. Data are shown as mean ± SEM from three biologically independent experiments.

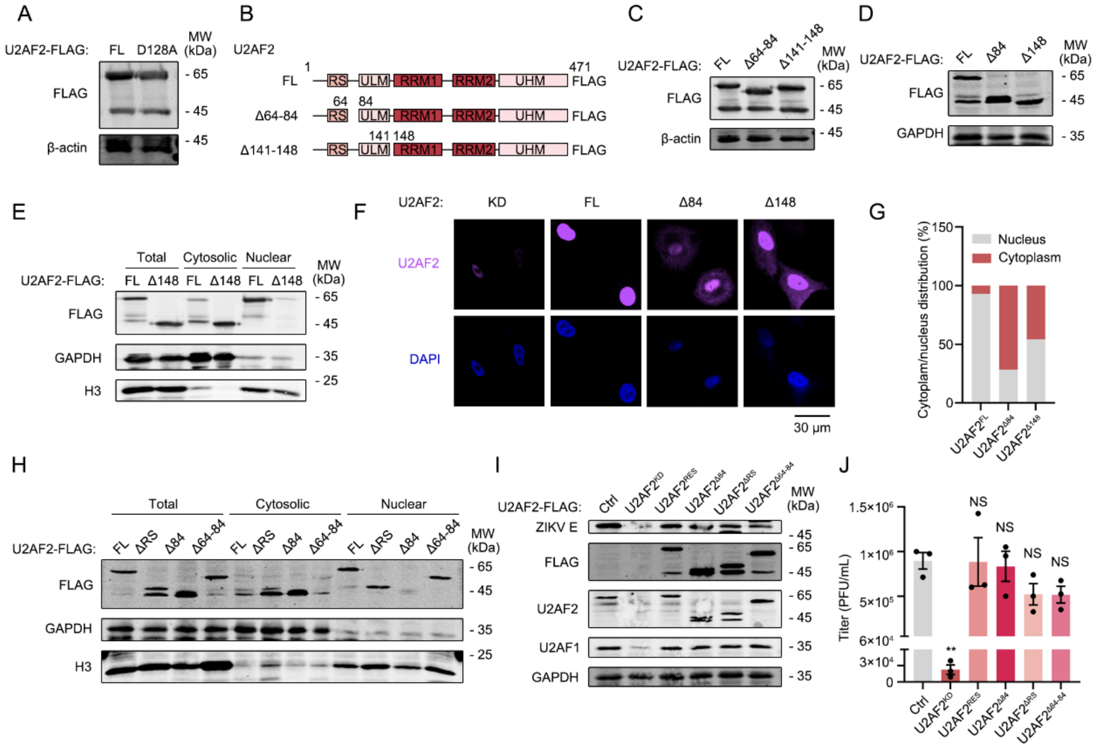

Fig. S6. Characterization of the cytoplasmic forms of U2AF2. (A) Western blot to detect expression level of U2AF2 D128A mutant. Cells were harvested for western blot using anti-Flag antibody. Representative images of three independent experiments are shown. (B) Schematic representation of full-length and two truncated U2AF2 proteins. (C-D) Western blot to detect expression levels of U2AF2 truncates. Cells expressing U2AF2 truncates (U2AF2$^{\Delta 64\text{-}84}$, U2AF2$^{\Delta 141\text{-}148}$, U2AF2$^{\Delta 84}$, or U2AF2$^{\Delta 148}$) were harvested for western blot using anti-Flag antibody. Representative images of three independent experiments are shown. (E-H) Subcellular distributions of U2AF2 full-length and truncated forms. Cells expressing U2AF2^{FL} and U2AF2$^{\Delta 148}$, or U2AF2$^{\Delta RS}$, U2AF2$^{\Delta 84}$, and U2AF2$^{\Delta 64\text{-}84}$ were harvested for subcellular fractionation (E, H). Cytoplasmic and nuclear fractions were subjected to western blot using anti-Flag antibody. GAPDH or H3 served as internal control for cytoplasmic or nuclear fractions. Representative images of three independent experiments are shown. Cells expressing U2AF2^{FL}, U2AF2$^{\Delta 84}$, and U2AF2$^{\Delta 148}$ were collected for IFM assay using U2AF2 antibody (F). The cytoplasmic and nuclear staining signals were quantified by Image J. (I-J) Detection of viral replication levels. Control, U2AF2^{KD}, U2AF2^{RES}, U2AF2$^{\Delta 84}$, U2AF2$^{\Delta RS}$, and U2AF2$^{\Delta 64\text{-}84}$ cells were infected with ZIKV at an MOI of 3. At 24 h p.i., cells and supernatants were harvested for Western blot (I) and plaque assay (J). Statistical significance was determined using one-way ANOVA with Dunnett's multiple comparison test (**$p < 0.01$ and NS, not significant). Data are shown as mean ± SEM from three biologically independent experiments.

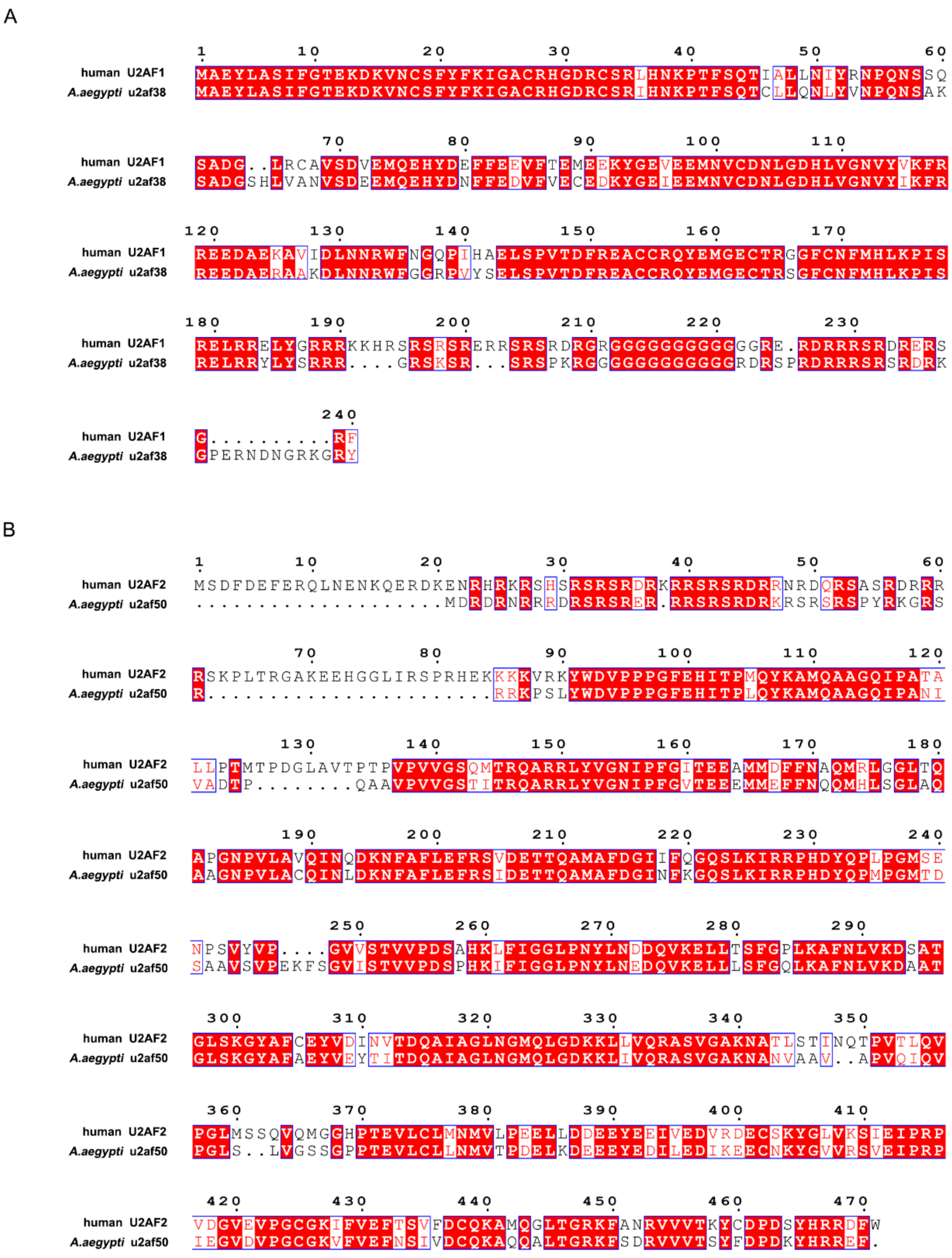

Fig. S7. Alignment of human protein U2AF1 and U2AF2 with their mosquito homologs. (A) Sequence alignment of human U2AF1 (NP_006749) with its homologs u2af38 (XP_001657976) from *A. aegypti*. (B) Sequence alignment of human U2AF2 (NP_001012496) with its homologs u2af50 (XP_001662443) from *A. aegypti*. Alignment was performed using DNAMAN and ESPript 3.0[46].

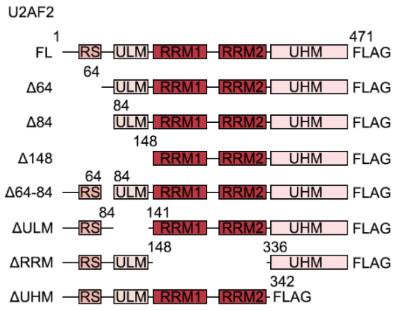

U2AF2	Cellular sublocalization	Cytoplasmic fragment (45 kDa)	Proviral activity
Full Length	Nucleus	Intact	+
Δ64(ΔRS)	Nucleus	Intact	+
Δ84	Cytoplasm	Intact	+
Δ148	Cytoplasm	< 45 kDa	-
Δ64-84	Nucleus	Intact	+
ΔULM	N.D.	< 45 kDa	-
ΔRRM	N.D.	< 45 kDa	-
ΔUHM	N.D.	< 45 kDa	-

Fig. S8. Summary of U2AF2 regions mediating its subcellular localization and proviral function.

Supplemental Tables

Table S1 Sequences of primers used in gene editing

Gene	Primers	Sequence (5'-3')
TGM2	sgRNA-5F	CACCGCCCAAGTTCCTGAAGAACGC
	sgRNA-3R	AAACGCGTTCTTCAGGAACTTGGGC
U2AF2	sgRNA-5F	CACCGCCCGGGACAGGCGACGACGC
	sgRNA-3R	AAACGCGTCGTCGCCTGTCCCGGGC
ATP6V1B2	sgRNA-5F	CACCGTGGTTCCAAGGCAGTAGTTC
	sgRNA-3R	AAACGAACTACTGCCTTGGAACCAC
APEH	sgRNA-5F	CACCGGAGCTTCAACCTGTCAGCGC
	sgRNA-3R	AAACGCGCTGACAGGTTGAAGCTCC
EFTUD2	sgRNA-5F	CACCGACCTGTTACGGTGTATGAGA
	sgRNA-3R	AAACTCTCATACACCGTAACAGGTC
MCM2	sgRNA-5F	CACCGAACCAGCTGATCCGCACCAG
	sgRNA-3R	AAACCTGGTGCGGATCAGCTGGTTC
XRCC5	sgRNA-5F	CACCGGGGGAAGTGCTTCTCTGTTT
	sgRNA-3R	AAACAAACAGAGAAGCACTTCCCCC
IDH1	sgRNA-5F	CACCGATAACCTACACACCAAGTGA
	sgRNA-3R	AAACTCACTTGGTGTGTAGGTTATC
RUVBL1	sgRNA-5F	CACCGGCCTCCCACAGCCACGTGAA
	sgRNA-3R	AAACTTCACGTGGCTGTGGGAGGCC
PHGDH	sgRNA-5F	CACCGTTGGGAGAGAGGTAGCTACC
	sgRNA-3R	AAACGGTAGCTACCTCTCTCCCAAC
LDHA	sgRNA-5F	CACCGAGCCCGATTCCGTTACCTAA
	sgRNA-3R	AAACTTAGGTAACGGAATCGGGCTC
CAP1	sgRNA-5F	CACCGAGGAGCAGCTCCATATGTGC
	sgRNA-3R	AAACGCACATATGGAGCTGCTCCTC
NUDC	sgRNA-5F	CACCGAGAAGAAGGGATGGCAGAGA
	sgRNA-3R	AAACTCTCTGCCATCCCTTCTTCTC
SLC18A2	sgRNA-5F	CACCGTGCAGGAGAGCCGCCGCTCG
	sgRNA-3R	AAACCGAGCGGCGGCTCTCCTGCAC
GARS1	sgRNA-5F	CACCGAGTAGACGTAGACAAAGCAG
	sgRNA-3R	AAACCTGCTTTGTCTACGTCTACTC
RAB14	sgRNA-5F	CACCGACAAGAATAATCGAAGTTAG
	sgRNA-3R	AAACCTAACTTCGATTATTCTTGTC
PSMD13	sgRNA-5F	CACCGAGCTCTAAAATTAAACATCG
	sgRNA-3R	AAACCGATGTTTAATTTTAGAGCTC
WDR1	sgRNA-5F	CACCGAGGGGCGTCTCCAAGATCAT
	sgRNA-3R	AAACATGATCTTGGAGACGCCCCTC
M6PR	sgRNA-5F	CACCGAACGAGACTCACATCTTCAA
	sgRNA-3R	AAACTTGAAGATGTGAGTCTCGTTC
U2AF1	sgRNA-5F	CACCGCGCCGAGCTGTCACCCGTGA
	sgRNA-3R	AAACTCACGGGTGACAGCTCGGCGC

U2AF2	shRNA-5F	CCGGACCCAACTACCTGAACGATGACTCGA GTCATCGTTCAGGTAGTTGGGTTTTTG
	shRNA-3R	AATTCAAAAAACCCAACTACCTGAACGATG ACTCGAGTCATCGTTCAGGTAGTTGGGT

Table S2 Sequences of primers used in cloning

Primers	Sequence (5'-3')	
U2AF1-CDS	5F	CGCGGATCCATGGCGGAGTATCTGGCCTC
	3R	CGGAATTCTCACTTATCGTCGTCATCCTTGTAATC ACCGAATCGCCCAGATCTTTCAC
U2AF1-RES-sgRNA	5F	TAATGGACAGCCGATCCATGCGGAATTAAGCCCG GTCACCGACTTCAGAGAAGCCT
	3R	AGGCTTCTCTGAAGTCGGTGACCGGGCTTAATTC CGCATGGATCGGCTGTCCATTA
U2AF1-ΔZn1	5F	CGCGGATCCATGACGTTTAGCCAGACCATCTTGA T
U2AF1-ΔRRM	5F	TCAAAACTCTTCCCAGTCTGCTGACGGTACCGACT TCAGAGAAGCCTGCTGCCGTCA
	3R	TGACGGCAGCAGGCTTCTCTGAAGTCGGTACCGT CAGCAGACTGGGAAGAGTTTTGA
U2AF1-ΔZn2	5F	CATGCGGAATTAAGCCCGGTCACCATTTCCAGAG AGCTGCGGCGGGAG
	3R	CTCCCGCCGCAGCTCTCTGGAAATGGTGACCGGG CTTAATTCCGCATG
U2AF1-ΔRS	3R	CGGAATTCTCACTTATCGTCGTCATCCTTGTAATC ACCCCGCAGCTCTCTGGAAATGGG
U2AF2-CDS	5F	CGCGGATCCATGTCGGACTTCGACGAGTTC
	3R	CGGAATTCCTACTTATCGTCGTCATCCTTGTAATC ACCACCCCAGAAGTCCCGGCGGTG
U2AF2-RES-shRNA	5F	AGCTGTTCATCGGGGGCTTGCCGAATTATTTAAAT GACGACCAGGTCAAAGAGCTGCTG
	3R	CAGCAGCTCTTTGACCTGGTCGTCATTTAAATAAT TCGGCAAGCCCCCGATGAACAGCT
U2AF2-ΔRS	5F	CGCGGATCCATGCCTTTGACCAGAGGCGCTAAA
U2AF2-ΔULM	5F	GATTCGTTCCCCCCGCCACGAGAAGGGGAGCCAG ATGACCAGACAAGCCC
	3R	GGGCTTGTCTGGTCATCTGGCTCCCCTTCTCGTGG CGGGGGGAACGAATC
U2AF2-ΔRRM	5F	GGTCGGGAGCCAGATGACCAGACAAGTGGGAGC CAAGAATGCCACGCTGA
	3R	TCAGCGTGGCATTCTTGGCTCCCACTTGTCTGGTC ATCTGGCTCCCGACC
U2AF2-ΔUHM	3R	CGGAATTCCTACTTATCGTCGTCATCCTTGTAATC ACCGGCATTCTTGGCTCCCACACT

U2AF2-D128A	5F	CACTGCTCTTCTCCCCACCATGACCCCTGCCGGTC TGGCTGTGACCCCAACGCCGGTGC
	3R	GCACCGGCGTTGGGGTCACAGCCAGACCGGCAGG GGTCATGGTGGGGAGAAGAGCAGTG
U2AF2-Δ64-84	5F	CCTCCCGGGACAGGCGACGACGCAGCAAAAAGA AGAAGGTCCGTAAATACTGGGACGT
	3R	ACGTCCCAGTATTTACGGACCTTCTTCTTTTTGCT GCGTCGTCGCCTGTCCCGGGAGG
U2AF2-Δ141-148	5F	CTGTGACCCCAACGCCGGTGCCCGTGGTCCGGCG CCTCTACGTGGGCAACATCCCCTT
	3R	AAGGGGATGTTGCCCACGTAGAGGCGCCGGACCA CGGGCACCGGCGTTGGGGTCACAG
U2AF2-Δ84	5F	CGCGGATCCATGAAGAAGAAGGTCCGTAAATACT
U2AF2-Δ148	5F	CGGATCCATGTACCCATACGATGTTCCAGATTAC GCTGGTCGGCGCCTCTACGTGGGCA
U2AF2-W92A	5F	GAAGAAGAAGAAGGTCCGTAAATACGCGGACGT GCCACCCCCAGGCTTTGAGC
	3R	GCTCAAAGCCTGGGGGTGGCACGTCCGCGTATTT ACGGACCTTCTTCTTCTTC
pFK-5'UTR	5F	GAATTCGGCCGGCATGGTCCCAGCCTCCT
	3R	GAATTCGACCAGAAACTCTCGTTTCCAAA
pFK-3'UTR	5F	GAATTCGCACCAATCTTAATGTTGTCAGG
	3R	GAATTCCTATAGTGAGTCGTATTACCTGC

Table S3 Sequences of primers used in qRT-PCR

Gene	Sequence (5'-3')	
TGM2	5F	CCAACTACAACTCGGCCCAT
	3R	CTGGTCATCCACGACTCCAC
U2AF2	5F	CGGCAGCTCAACGAGAATAAA
	3R	GGGAACGAATCAGTCCACCG
ATP6V1B2	5F	AGTCAGTCGGAACTACCTCTC
	3R	CATCCGGTAAGGTCAAATGGAC
APEH	5F	CCCCATTCATCCTTTGTCAC
	3R	AAAGCCCATCTTGCAAAGC
EFTUD2	5F	CAATATCATGGACACTCCAGGAC
	3R	CGGTCAATCTTGTTGATGCACA
MCM2	5F	GCCAAGATGTACAGTGACCTGA
	3R	GATGTGCCGCACCGTAAT
XRCC5	5F	TGACTTCCTGGATGCACTAATCGT
	3R	TTGGAGCCAATGGTCAGTCG
IDH1	5F	TGTGGTAGAGATGCAAGGAGA
	3R	TTGGTGACTTGGTCGTTGGTG
RUVBL1	5F	GGACATCACATCCCCTCACG
	3R	GCTGCACTGAGTACCTGTTTC

PHGDH	5F	TACCACAGGCTTGCTGAATGA
	3R	GAGCACAGTTCACTACTCGCA
LDHA	5F	ATGGCAACTCTAAAGGATCA
	3R	GCAACTTGCAGTTCGGGC
CAP1	5F	ATGCACCGTGGGTATGCAG
	3R	AAGCAGCGAGTCAAATGCCT
NUDC	5F	GTGACTGTGCATCTGGAGAAG
	3R	CTTGGTGTTGATCTCAGGGTC
SLC18A2	5F	CCTGAATGAAAACGTGCAAGTTG
	3R	AGTAGTCCTATGAAAGGGTTGGT
GARS1	5F	AAAGCCCGCAAGAGGGTTC
	3R	CTTCCATTTTTGCTCGGTCTACA
RAB14	5F	GAAAATGGCTTATTGTTCCTCG
	3R	GGGTTGGGGTTCACTGGTTAG
PSMD13	5F	CAGATGACTGATCCTAATGTGGC
	3R	CCAGGAAGGTTGTTGAGCATTT
WDR1	5F	CGGGTACATCAACTATCTGGACA
	3R	GTTTTTATGCACCGTCAGACAC
M6PR	5F	CTGGAGGACTGGACTGCTACT
	3R	CTCCTACCAAGTCGCAAGTTTT
U2AF1	5F	AGTAGAGGAGATGAACGTCTGTG
	3R	GGATCGGCTGTCCATTAAACC
ZIKV NS1	5F	GTCAGAGCAGCAAAGACAA
	3R	CAGCCTCCTTTCCCTTAACA
H3	5F	GCTACCAGAAGTCCACGGAG
	3R	GATGTCCTTGGGCATAATGG
SEC61α	5F	CTATTTCCAGGGCTTCCGAGT
	3R	AGGTGTTGTACTGGCCTCGGT
Bip	5F	ACTTGGGGACCACCTATTCCT
	3R	ATCGCCAATCAGACGCTCC
β-actin	5F	GCTCCTCCTGAGCGCAAG
	3R	CATCTGCTGGAAGGTGGACA
HMGCR	5F	TTCTTGCCAACTACTTCGTGTT
	3R	GCTGCCAAATTGGACGACC
SARS-CoV-2	5F	CGAAAGGTAAGATGGAGAGCC
	3R	TGTTGACGTGCCTCTGATAAG
Aegypti-u2af38	5F	AATGCACTCGGTCGGGATTC
	3R	AACGCTTTCGCCTCAGTATCT
Aegypti-u2af50	5F	GATCTTCATCGGCGGTTTGC
	3R	AACGTTTGCGTTCTTGGCTC
Aegypti-actin	5F	GAACACCCAGTCCTGCTGACA
	3R	TGCGTCATCTTCTCACGGTTAG

Table S4 Sequences of primers used in dsRNA synthesis

Gene	Sequence (5'-3')	
GFP	5F	TAATACGACTCACTATAGGCAACAGCCACAACG TCTATATC
	3R	TAATACGACTCACTATAGGTGATCCCGGCGGCG GTCACGAA
Aegypti-u2af38	5F	TAATACGACTCACTATAGGTCGCCGGTGACCGA CTTTCGCG
	3R	TAATACGACTCACTATAGGTCCCTTGCGATCGCG GCTTCGA
Aegypti-u2af50	5F	TAATACGACTCACTATAGGGTTGCGGCTGTCGC ACCAGTTC
	3R	TAATACGACTCACTATAGGAATTGAGTTAAATTC GACAAAG

Table S5 Primary antibodies used in western blot and IFM

Antibody	Source	Identifier
U2AF1 mouse monoclonal Ab	Santa Cruz	sc-514459
U2AF2 mouse monoclonal Ab	Santa Cruz	sc-53942
ZIKV E rabbit polyclonal Ab	GeneTex	GTX133314
β-actin mouse monoclonal Ab	Sigma	A1928
PAPR rabbit monoclonal Ab	CST	9532
H3 rabbit polyclonal Ab	Proteintech	17168-1-AP
FLAG-tag polyclonal Ab	MBL	PM020
GAPDH rabbit monoclonal Ab	Proteintech	10494-1-AP
Calnexin rabbit polyclonal Ab	Proteintech	10427-2-AP
EEA1 mouse monoclonal Ab	Proteintech	68065-1-LP
Lamin B1 rabbit monoclonal Ab	Proteintech	12987-1-AP

Table S6 List of proteins enriched in the ER of ZIKV- and DENV2-infected cells

Gene	Accession	Foldchange	Unique peptides
SARS1	KAI2518243.1	10.82	4
HNRNPF	KAI2555588.1	8.15	5
CORO1B	NP_065174.1	7.65	3
AMPD2	KAI4081835.1	7.29	1
AL4A1	NP_001306147.1	6.99	2
TGM2	NP_001310245.1	6.61	11
U2AF2	KAI4044995.1	6.54	5
GNAI3	KAI2518279.1	6.44	3
ATP6V1B2	NP_001684.2	6.38	5
ANXA1	KAI4007414.1	6.26	5
IMPDH1	NP_001397690.1	6.21	19
TUBB	UQL50333.1	6.16	4
RTCA	NP_003720.1	5.95	1
AP1B1	NP_001365495.1	5.93	1

GNPDA1	NP_005462.1	5.89	3
HNRNPA0	NP_006796.1	5.76	1
APEH	XP_005265154.1	5.71	7
TCP1	NP_110379.2	5.62	22
PRMT1	KAI2592355.1	5.59	6
EFTUD2	NP_001136077.1	5.41	11
AHCY	XP_047295918.1	5.37	20
MCM2	XP_024309299.1	5.22	10
CCT6A	BAD92965.1	5.04	21
HNRNPA1	KAI4066325.1	5.01	10
HNRNPK	NP_001305115.1	4.99	22
XRCC5	KAI2526849.1	4.92	29
MVD	XP_011521390.1	4.81	5
TUBB4B	KAI2554760.1	4.67	1
ANXA4	KAI4034873.1	4.58	24
unnamed protein product	BAF83296.1	4.52	11
GOT1	KAI4077084.1	4.51	3
CAPN1	KAI4072231.1	4.44	3
CCT2	KAI2566966.1	4.31	23
HSPA1A	KAI2538859.1	4.21	20
NPEPPS	KAI2583659.1	4.16	5
RAN	KAI2568723.1	4.09	5
unnamed protein product	BAG35388.1	4.05	9
unnamed protein product	BAG35210.1	4.05	2
IDH1	KAI4037869.1	4.02	18
EIF4A1	NP_001407.1	3.95	11
MT-RNR2	XP_016876050.1	3.93	5
HNRNPU	KAI2591193.1	3.93	4
ALDH7A1	KAI2538643.1	3.91	2
PCBP1	NP_006187.2	3.90	6
PHGDH	KAI2518638.1	3.80	23
RUVBL1	NP_003698.1	3.80	17
unnamed protein product	BAG36947.1	3.77	13
DDX15	KAI4025039.1	3.74	16
CTSD	KAI2558045.1	3.66	9
LDHA	KAI4070363.1	3.63	21
CAP1	KAI4079966.1	3.63	19
DDX5	KAI4051133.1	3.63	8
NUDC	NP_006591.1	3.60	6
SLC18A2	KAI4049731.1	3.59	10
CSE1L	NP_001349691.1	3.58	18
UBA1	KAI3999419.1	3.57	23
CLTC	KAI2584308.1	3.55	52
ACY1	KAI4029990.1	3.55	3

FDPS	KAI4083049.1	3.52	3
ACAT2	NP_005882.2	3.49	12
unnamed protein product	BAG35102.1	3.47	7
GARS1	KAI4013318.1	3.41	20
GAPDHS	KAI2563954.1	3.38	32
PARP1	KAI4085156.1	3.37	6
AP1G1	KAI4055956.1	3.34	13
VPS35	KAI2578368.1	3.33	10
unnamed protein product	BAG35480.1	3.26	20
CLIC1	KAI2541674.1	3.21	4
RAB14	KAI4008255.1	3.18	9
OLA1	NP_001315617.1	3.17	4
unnamed protein product	BAG36219.1	3.15	4
S100P	KAI2533644.1	3.15	1
unnamed protein product	BAF84244.1	3.14	6
RAB32	AAM21106.1	3.14	3
RUVBL2	NP_006657.1	3.09	11
SEC23A	KAI2571044.1	3.07	11
HNRNPR	NP_005817.1	3.05	5
COPS3	NP_001303284.1	3.04	1
PSMD13	NP_002808.3	3.04	6
LUC7L	KAI2576099.1	3.03	1
XRCC6	KAI4003213.1	3.00	29
unnamed protein product	BAF82041.1	2.99	8
unnamed protein product	AAF19249.1	2.99	5
unnamed protein product	BAG37316.1	2.98	12
unnamed protein product	BAG35186.1	2.96	8
PSMD11	KAI4048806.1	2.92	7
HSPH1	XP_016875852.1	2.9	17
WDR1	KAI4024872.1	2.88	20
RACK1	NP_006089.1	2.86	18
CELF1	KAI4071109.1	2.85	1
RAD23B	KAI4007982.1	2.84	4
ACTB	KAI4012802.1	2.83	14
unnamed protein product	AAC23787.1	2.81	2
HSP90AB1	KAI4062471.1	2.80	28
unnamed protein product	CAG30336.1	2.80	13
EIF6	NP_852133.1	2.78	3
VCP	NP_001341856.1	2.76	43
M6PR	KAI4064575.1	2.76	6
GSTP1	KAI4072669.1	2.74	2
IPO7	NP_006382.1	2.71	9
unnamed protein product	BAG37806.1	2.65	20
SULT2A1	NP_003158.2	2.65	1

ALDOA	KAI4054480.1	2.58	22
VCL	NP_003364.1	2.56	14
HSPA1B	KAI2541696.1	2.53	3
DNM2	KAI2588733.1	2.52	6
NQO1	KAI2579412.1	2.51	3
unnamed protein product	AAY40954.1	2.50	12
unnamed protein product	BAF84650.1	2.49	26
unnamed protein product	BAG35185.1	2.48	38
ACSL3	NP_001341087.1	2.46	16
OSBP	KAI2560135.1	2.42	15
FARSB	KAI4038343.1	2.40	11
CPNE3	XP_047278350.1	2.40	6
HSP90AB1	NP_001258901.1	2.39	34
STX7	KAI2543864.1	2.39	7
GFPT1	KAI2523707.1	2.37	23
PYGB	NP_002853.2	2.37	11
YWHAB	KAI2594960.1	2.35	3
UPF1	XP_047295147.1	2.34	12
NOP56	KAI2593880.1	2.32	6
TUBB2A	NP_001060.1	2.31	2
DDX1	NP_004930.1	2.28	19
MARS1	KAI2566573.1	2.28	10
KIF5B	KAI4075630.1	2.28	12
PSMD2	KAI2532741.1	2.27	11
HSPB1	KAI4014259.1	2.27	5
RPL13A	KAI2592279.1	2.24	13
H4C1	KAI2564581.1	2.22	2
CTSA	NP_001161066.2	2.18	2
MOV10	NP_001376491.1	2.12	24
ALDH1A1	NP_000680.2	2.09	16
unnamed protein product	BAF85493.1	2.06	4
PRDX2	KAI4040726.1	2.05	6
STRAP	NP_009109.3	2.04	8
